# Fidelity to territory and mate and the causes and consequences of breeding dispersal in American goshawk (*Astur atricapillus*)

**DOI:** 10.1371/journal.pone.0323805

**Published:** 2025-05-22

**Authors:** Richard T. Reynolds, Shannon L. Kay, Jeffrey S. Lambert, Martha Ellis

**Affiliations:** 1 United States of America Department of Agriculture, Forest Service, Rocky Mountain Research Station, Fort Collins, Colorado, United States of America; 2 Montana State University, Bozeman, Montana, United States of America; UFERSA: Universidade Federal Rural do Semi-Arido, BRAZIL

## Abstract

Using mark-resight data, we investigated fidelity to territory and mate as well as breeding dispersal rates and the causes and consequences of breeding dispersal in a 20-year study of American goshawks (*Astur atricapillus*) in Arizona, USA. Generalized Additive Mixed Models were used to identify the relative contributions of four prominent explanatory variables (eggs laid, nest failed, nest successful, mate loss) and 21 individual and environmental variables in a machine learning Conditional Inference Forest to predict breeding dispersal. Ninety-five percent of males and 92% of females exhibited lifetime territory fidelity and 97% exhibited lifetime mate fidelity. Mate loss alone (to divorce, possible emigration or death) made the biggest difference in the predicted probability of dispersal (0.11 with mate loss, 0.005 with mate retention). Yet, in 80% of mate losses a hawk stayed on its territory to eventually nest with a new mate. Territory fidelity was highest when the mate was retained in the next breeding and the pair’s previous attempt produced fledglings. All males and 86% of females that dispersed to a territory in our study area moved no farther than to a 3rd-order neighboring territory (crossed 2 territories). Despite equivocal evidence of dispersal to territories more frequently occupied by egg-layers, there was otherwise little evidence that hawks on average dispersed to better territories. On average reproduction did not improve post-dispersal and dispersers did not move to territories with greater total (all monitored yrs) reproduction. Goshawks losing their mates appeared to use a home-based mate searching that minimized loss of a familiar territory by waiting on their territory for a new mate and prospecting nearby territories for unpaired mates. The small sample of nearby prospected territories, combined with fortuitous occurrences of unpaired mates, resulted in random (with respect to quality) selections of territories by dispersers.

## Introduction

The frequency of dispersal and dispersal distances are fundamental to source-sink and meta-population dynamics [1,2], invasion and colonization [3], gene flow and population genetic structure [4], species interactions [5], habitat selection [6], and conservation of species and their habitats [7,8]. Breeding dispersal, moving from one breeding territory to another [9], is thought to be a reproductive strategy whereby individuals improve their fitness by moving to higher quality territories and/or mates [10,11]. Though some birds had improved fitness post-dispersal [12–15], others did not, showing that breeding dispersal can be non-adaptive or neutral [16–22]. Benefits of dispersal include avoiding a missed breeding season after loss of mate [13], escaping predation, competition, increased breeding success, and temporally varying resource conditions [23,24]. Costs of breeding dispersal include increased mortality risks [25,26], loss of a familiar territory [27,28], missing a breeding season when better opportunities cannot be found [29,30], uncertain qualities of new territories or mates, and increased energy expenditure during searches for a new territory or mate [31]. Costs and benefits depend on the availability and quality of alternative territories or mates and the availability and quality of information for assessing the alternatives [32], reviewed in [33].

The flip side of breeding dispersal, fidelity to territory, is common in both migratory and non-migratory birds [34], and mate fidelity is particularly common in long-lived species with year-round pair bonds [35]. Territory fidelity has been reported to increase with age, breeding experience, and familiarity with a territory [11,36–40]. Advantages of faithfulness to territory and mate are familiarity with resource locations such as food, nest sites, predators, neighboring conspecifics, increased efficiency in territory defense [27,38], and improved mate coordination [39–41]. Decisions to stay or disperse involve trade-offs between costs and benefits and involve multiple choices: when to change territories, how far to move, and on which territory and with which mate to settle [13,34,42–45].

The frequency of breeding dispersal and distance moved may be influenced by habitat and a population’s demography [42–44]. Individuals occupying low-quality habitats have been reported to disperse more often and farther, and dispersal distances may be longer in patchy than in contiguous habitat where territory density can be higher [24,42,46–48]. Alternatively, competition for territories and mates in saturated habitats may be so intense that attempted dispersal may result in loss of territory or mate leading to reduced fitness [34].

The probability of breeding dispersal in raptors has been associated with sex, age, body condition, breeding failure, territory and/or mate quality, territory familiarity, mate loss, divorce, predation, competition, and conspecific competitive ejection [11,43,46,49–54]. In many monogamous birds, males disperse less frequently and move shorter distances than females, the theoretical consequence of a male’s greater costs of giving up familiar territories as males typically defend territories and are the primary food provider during breeding [55]. Among raptors, young breeders often disperse more frequently than older individuals, and both sexes tend to disperse short distances, often to neighboring territories [11,42,56], but see [13]. There remains, however, little information on the causes and consequence of breeding dispersal in some raptors because of difficulties of gathering longitudinal data, especially in long-lived species, and dispersal may occur over too large a spatial scale to detect individuals that move beyond study area boundaries [42,57,58].

Habitat quality is an important determinate of fitness in territorial species [59–61]. Measures of a territory’s quality have been based on the quantity and quality of resources such as food, availability of nest sites, the structure and composition of vegetation, predation risks, the frequency of occupation, the phenotypic characteristics of occupants such as body size or condition, seasonal sequential date of occupation, and the age, reproduction, and survival of breeders [23,62–69]. A frequently cited model of territory choice is the ideal despotic distribution (IDD) by breeding pairs wherein older, dominant individuals monopolize the best territories thereby relegating lower quality individuals (young, subordinate) to progressively poorer territories where reproduction is lower [64].

Given the importance of territory quality to individual fitness and conservation of quality habitats, a common objective of breeding dispersal studies is to identify its causes and its consequences on fitness [11,46,50,70–74], the cues used in choosing territories or mates [75–78], and whether territory changes followed the IDD model [65,70,79]. If a disperser’s territory choice manifests as movements up a habitat quality gradient (the IDD model), then breeding dispersal may aid the ranking of territories in studies of the vegetation composition and structure that confers habitat quality to a species [80]. Studies of breeding dispersal in large territorial birds – species that typically defend large territories spaced at long distances – require mark-resight protocols applied in large study areas. Thus, studies of breeding dispersal in large raptors (i.e., American goshawk) are more likely to be spatially limited with increased probability of underestimating the frequency and distances of dispersal due to beyond study area movements. Furthermore, because large raptors typically show high territory fidelity (and infrequent dispersal) and are long-lived, investigations into the causes and consequences of territory changes on a disperser’s lifetime fitness need to be of sufficient duration to document a suitable sample of dispersals. A few reports of territory fidelity and breeding dispersal frequency and distances moved in American goshawks exist, most based on relatively few territorial pairs in relatively short-term studies (see below). However, the causes of dispersal, the factors affecting which territory to settle in, and the reproductive consequences of the choices have not been investigated.

To determine the extent of fidelity to territory and mate and the correlates and consequence of breeding dispersal and divorce in the American goshawk (*Astur atricapillus*) [formally the northern goshawk (*Accipiter gentilis atricapillus* [82]) [81,82], we used a 20-yr (1991–2010) mark-resight data set on color-banded individuals in a large sample of breeding territories (up to 125) on the Kaibab Plateau, Arizona, USA [83,84]. Our objectives were: (1) to document territory and mate fidelity rates, breeding dispersal rates, and dispersal distances, (2) to investigate factors that affect not only the decision to change territories but which territory to settle in, and (3) to investigate the consequences of dispersal on a goshawk’s reproductive fitness. We first tested the hypothesis that breeding dispersal in goshawks was caused by one of the leading predictors of dispersal in raptors (i.e., breeding failure, sex, age, territory familiarity, and mate loss) with a generalized additive mixed model (GAMM). In a wider search of predictors, we investigated 21 potential individual and environmental variables (inclusive of the GAMM predictors) that predicted breeding dispersal with a supervised machine-learning, conditional inference forest (CIF). We next tested the hypothesis that dispersals followed predictions of the IDD model of habitat selection; that is, whether dispersal was adaptive, neutral, or non-adaptive, by comparing (1) the total (all monitored yrs) territory occupancy rate by egg-laying pairs in a disperser’s original and new territory (2) a disperser’s reproductive performance before and after dispersal, (3) a disperser’s total fledgling production during its tenure in its original vs. the total fledglings produced by breeders in the disperser’s new territory during those same tenured years (when a potential disperser could appraise breeding attempts during prospecting intrusions into the new territory), and (4) the total fledglings produced by the ensemble of breeders in a disperser’s original vs. new territory. We also report the divorce rate and investigated whether it was adaptive by comparing the reproductive performance of divorced males and females on their original vs. their new territory and/or mate.

## Methods

### Study area

**Fig 1:**
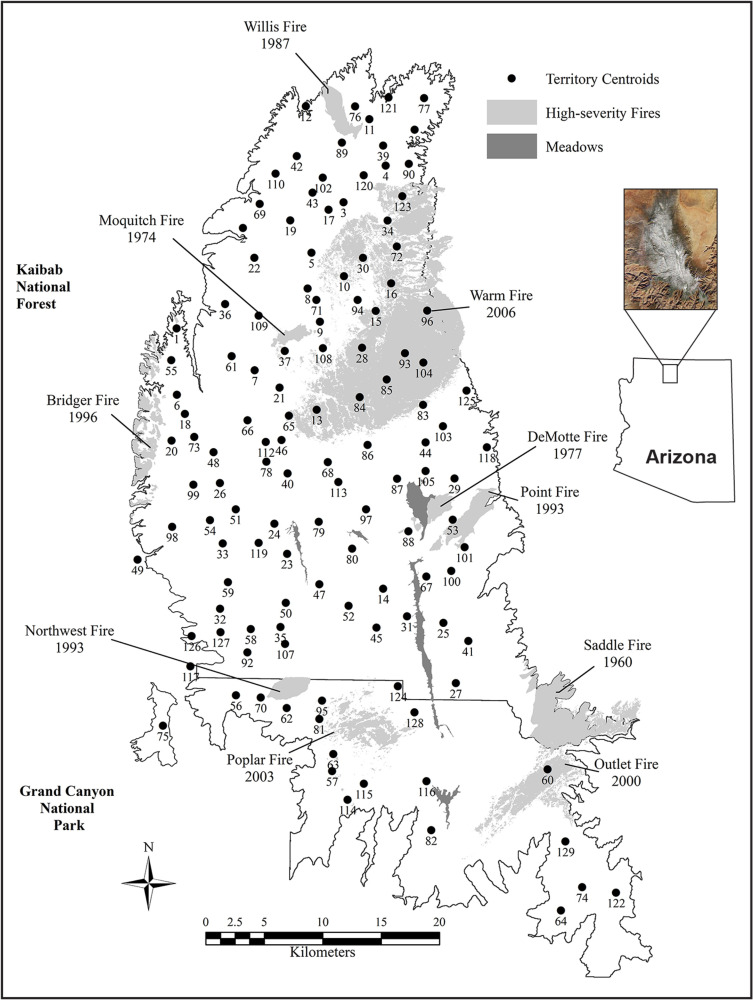
The Kaibab Plateau study area encompassing goshawk breeding territories in northern Arizona, USA. The 1,728-km^2^ study area included the entirety of the Kaibab Plateau above 2,182 m above sea level. The southern one-third of the study area included the Grand Canyon National Park-North Rim, and the northern two-thirds included the North Kaibab Ranger District of the Kaibab National Forest. Displayed are the numbered centroids (see Methods) of 125 American goshawk breeding territories, natural meadows, and the location and year of high-severity fires. Territory numbers in the figure correspond to territory numbers displayed in S1 Fig S1 Appendix. Map outline derived from Landsat 5 Thematic Mapper imagery courtesy of the U.S. Geological Survey, and the photo image provided courtesy of NASA/GSFC/LaRC/JPL, 12/31/2000 (photo image taken before the 2006 Warm Fire).

Our 1,728 km^2^ study area included all of the Kaibab Plateau above 2,182 m above sea level (a.s.l.) in northern Arizona, USA (36°29'29ʺN, 112°12'30ʺW), which included the North Kaibab Ranger District (NKRD) of the Kaibab National Forest and the Grand Canyon National Park-North Rim (GCNP-NR) (Fig 1). The study area had a maximum north–south and east–west length of 78 x 33 km, respectively. The Kaibab Plateau, a forested ‘island’, is an oval-shaped (~95 km x 55 km) limestone plateau rising to an elevation of 2,797 m a.s.l. from a surrounding shrub-steppe plain at ~1,700 m a.s.l. The Kaibab Plateau is isolated from other forests by varying expanses of a low elevation shrub-steppe plain (see below and Fig 1). The Plateau is dissected by moderately sloping drainages and is bounded by escarpments of the Grand Canyon of the Colorado River on its south side, steep slopes on the east side, and gentle slopes on both the north and west sides that descend to the plain. Forests at the highest elevation (>2,600 m a.s.l.) comprised a mesic and relatively dense mixed-conifer forest dominated by spruce (*Picea* spp*.*), fir (*Abies* spp*.*), Douglas-fir (*Pseudotsuga menziesii*), quaking aspen clones (*Populus tremuloides*), and patches of ponderosa pine (*Pinus ponderosa*) on ridge tops and south-facing slopes. A more open, dry mixed-conifer forest, dominated by ponderosa pine, Douglas-fir, white fir (*Abies concolor*), blue spruce (*Picea pungens*), and small aspen clones occurred at mid-elevations (2,450–2,600 m a.s.l.). Forests of nearly pure ponderosa pine occurred in the lowest elevation zone of the study area (2,075–2,450 m a.s.l.), but contained patches of mixed Douglas-fir, spruce, and aspen in drainages. Below the study area, a xeric, short-statured woodland of mixed pinyon pine (*Pinus* spp.) and juniper (*Juniperus* spp.) occurred at 1,700–2,182 m a.s.l. These woodlands extended up elevation into the ponderosa pine on south-facing slopes while ‘stringers’ of ponderosa pine extended down-drainages into the woodlands [83,85,86]. Desert scrubland occurred below 1,700 m a.s.l. (Fig 1). Except for several narrow (< 1 km wide) meadows, tree harvest areas (scattered blocks of 8–17 ha of shelterwood and/or seed-tree cuts, and single-tree harvests; [87]), and several areas burned by recent (since 1960) high-severity wildfire, forests on the Kaibab Plateau were continuous. A more complete description of the study area is in [88,89].

Except for a small area of ponderosa pine on the Grand Canyon-South Rim at 18 km from the southern boundary of our study area, distances to the nearest extensive forested areas were 90 km to the south (Coconino National Forest), 70 km to the west (Mount Trumbull), 97 km to the north (Dixie National Forest), and 250 km to the east (Chuska Mountains) [83]. Annual precipitation on the Kaibab Plateau averages 67.5 cm, with winter snowpack of 2.5–3.0 m [85]. Winters are cold and summers are cool. A drought period typically occurs from May to mid-July and is followed by a mid- to late-summer monsoon season with frequent (2–4 per week) thunderstorms and rain showers.

### Background and field methods

Goshawks are monogamous, territorial, typically lay one clutch annually [90], and are mostly non-migratory with breeding adults remaining on or near their territories in winter [90–94]; but see [95]. We monitored territory occupancy and reproduction by goshawks from April through September on as many as 125 territories from 1991–2010 [83,84]. A goshawk nest (and territory) was defined as active (i.e., eggs laid) when a nest was observed with an adult in incubation or brooding postures, or if eggs, nestlings, or fledglings were observed. Capturing and sexing breeding goshawks are described in [83,89]. All breeding goshawks received a USGS leg band and a colored aluminum band (blue for adult males, black for adult females) with a unique alpha-numeric code readable with 40–60 × telescopes and cameras. Use of two bands showed no band losses over the 20 years. Active nests were visited weekly to determine status, to count and band nestlings, and to determine the timing and cause of nest failure. Nestlings were banded with a USGS leg band and a colored band (green or orange) in the 10 days prior to fledging and counts of nestlings at banding were taken as the number of young fledged unless mortalities were noted prior to or within 5 days post-fledging.

We defined ‘territory’ as a nesting area exclusively occupied by one pair of nesting hawks during breeding. Territories were distributed in our study area in a regular pattern with a mean distance between territory centroids (see below) of 3.8 ± 0.1 km with a range of 1.2‒8.4 km (Fig 1). We estimated territory size (a circular area of ~11.3 km^2^) as the area whose radius was half (1.9 km) the 3.8 km mean distance between centers of first-order (the nearest) neighboring territories [83,89]. Annual occupancy of territories by egg-laying breeders was non-random, suggesting that some territories were preferentially occupied while others appeared to be avoided [84]. Based on the size of our study area, we estimated that it contained ~144 territories and that the final number of monitored territories (125) comprised ~87% the estimated 144 total. Mapped territories (Fig 1) showed that the NKRD portion of the study area was essentially saturated with territories and that most undiscovered territories were in the GCNP-NR where access was difficult logistically [83]. Each year a territory was classified as ‘occupied’ by egg-laying breeders if an active nest was found, ‘occupied-only’ if no active nest was found but adults were observed on ≥2 occasions in the vicinity of a nest or when a single adult was seen in a nest area that contained molted feathers, fresh feces, and new nest construction. Trapping, banding, and resighting of a hawk’s bands occurred almost exclusively in the immediate area of active nests. If no active nest was discovered in a territory by the completion of our annual intensive 3-step territory monitoring procedure (required 15–20 person-day nest searching visits per territory) to classify a territory as ‘status unknown’ given that an active nest may have been missed [89]. We defined a ‘failed nest’ as an active (eggs laid) nest from which all eggs or nestlings were lost. Mean annual active nest failure rate ranged from 0%–48% (mean = 22 ± 0.03%) and mean annual number of fledglings produced per successful (≥1 fledgling) nest was 1.87 ± 0.07, range = 1.46–2.43 fledglings). We defined a ‘prior nest attempt’ as a nest attempt (eggs laid) that preceded a focal nest attempt, whether it occurred in the previous calendar year or the last nest attempt prior to 1 or more years of missed egg-laying.

Fifty-eight males and 88 females were known-age recruits (banded as nestlings) to the local breeding population, and comprised 30% and 35%, respectively, of all banded breeders in our study [83]. All unbanded breeders at first capture (trapping occurred June to mid-July) were assigned to 1-year, 2-year, or 3-yr-old age-class based on extent of retained juvenal plumage and eye color, and to a ≥ 4-yr-old class if in full adult plumage [84]. Lifespan, age at first breeding, breeding lifespan, number of breeding attempts, and lifetime reproduction for individual banded goshawks are reported in [84]. All used nests within each territory were mapped with GPS, and territory ‘centroids’ were calculated as the geometric means of coordinates of all used alternates over multiple years weighted by the number of years each alternate was used. Mean distance from territory centroids to all within-territory alternate nests was 0.3 km with a maximum of 1.5 km [83]. The proportion of monitored territories with egg-laying breeders varied extensively; ~ 94% of territories had breeders with eggs in the best breeding year (1992) while only 9% of territories had eggs in the worst year (2003; see S1 Table in S2 Appendix, S1 Fig in S1 Appendix). Inter-annual variation in egg-laying tracked variations in prey abundance driven by El Niño-Southern Oscillation (ENSO) variation in precipitation that affected primary forest productivity that cascaded up through primary and secondary consumers, including goshawk prey [83,96]. We used the proportion of territories occupied by egg-laying pairs in a year as a measure of a year’s quality for breeding; in good breeding years more territories had egg-laying hawks, brood sizes were larger, and fewer nests failed [83].

We defined ‘breeding dispersal’ (*contra* movements to within-territory alternate nests [83]) as movements by breeders to nests >1.9 km (mean radius of territories) from their original territory’s centroid to a location within 1.9 km of a different territory’s centroid. We report two rectilinear dispersal distances: the distance from the last used nest in the original to the first used in a new territory, and the distance from the centroid of the original to the centroid of the new territory.

In poor breeding years territorial goshawks often skipped egg-laying and more nests failed before their band codes could be fully resighted. Our study of goshawk survival [83] found that annual mark-resight detection probabilities of breeding goshawks ranged from 0.85 in good breeding years to 0.12 in poor years (see S1 Fig in S1 Appendix). Low detection probabilities were the consequences of our nearly complete inability to resight the band codes of non-egg-laying and early failed breeders due their (presumably) within-home range movements after nest abandonment. When detection probabilities are low (<0.15) and the number of visits high (>15), it is infeasible for a mark-resight model of occupancy to distinguish between a territory where the hawk is absent and a territory in which the hawk simply went undetected [97]. Nonetheless, during multiple nest-searching visits to territories not confirmed to have an active nest, a goshawk would occasionally respond to broadcasts of their vocalizations with a fly-over or a brief perching. Because these quick visitors could not be resighted (even if banded) and because goshawks typically have overlapping home ranges and often make intrusions into neighboring territories [75,93,94], these “visitors” could not be counted as detections of the non-breeding territory owner. Instead of modeling detection probabilities to estimate territory occupancy in years of missed resights given their near zero detectability, we estimated a hawk’s territory fidelity (i.e., occupancy) in two ways. We first determined territory fidelity by only tallying a hawk’s consecutive years of egg-laying on the same territory (i.e., excluding yrs of missed resights). Second, we estimated a hawk’s fidelity as the number of consecutive years a banded breeder retained its territory + the number of years it was inferred to have retained its territory in missed resight years. We inferred a hawk’s territory retention only if the missed resight year was bracketed in the years before and after by resights of the same hawk on the same territory (hereafter, the bracketed rule).

Our bracketed rule was based on 5 validating factors: (1) *a priori* evidence of strong year-round goshawk territory fidelity [89,93,94], (2) the limited number of consecutive years over which retention inferences were made ($\overset{―}{x}$ = 1.4 ± 0.05; median = 1; range = 1–6 yrs), (3) only 3 dispersed hawks in the 20-year study moved back to their original territory 3–6 later, (4) the predominance of short dispersal distances (see below), which lowered the probability of missed dispersals, especially after the third study year (1993) when most of territories in our large study area were under intensive monitoring, and (5) no hawks whose territory retentions were inferred in a year were observed as dispersed in that year (see Results and Fig 1, S1 Fig in S1 Appendix). In a few cases of partially resighted band codes (bands observed but codes partially missed), we assumed a banded hawk had occupied its territory in that year if its band was the same color (blue, black, green, orange) and on the same leg only if the partial resight occurred in no more than two years before or after a complete reading of the hawk’s band, and the partial reads eliminated other potential dispersals by occupants of surrounding territories. We determined overall territory fidelity rates as equal to 1 minus the proportion of all banded hawks (sexes separately) that made breeding dispersals, and the overall dispersal rates as 1 minus the proportion of hawks that retained their territories.

We defined divorce (a cause of dispersal) as the separation of a breeding pair as evidenced by each pair member nesting with different mates in a subsequent year. We calculated the mate fidelity rate as the total proportion of surviving pairs that retained their mate one year to the next as equal to 1 minus the proportion of pairs that divorced, and the divorce rate as 1 minus the proportion of pairs that retained their mates [39,41]. As with dispersal, two mate fidelity and divorce rates were determined: first, we used only the count of consecutive year resights of mate retentions, and second, we used the combined count of consecutive years of known mate retentions with counts of years when the identity in missed resight years of one or both pair members was inferred to have retained their mates with the bracketed rule. We defined a disperser’s ‘familiarity’ with its original territory as the number of years a hawk was known or inferred to have been in its original territory and a disperser’s ‘breeding experience’ on its territory as the number of breeding attempts made in its original territory prior to a dispersal.

#### Ethics and animal welfare.

Capturing and banding of goshawks were conducted under United States Fish and Wildlife Service Banding and Auxiliary Marking permit (#21294), United States Geological Service Scientific Collecting permit (#MB044583–0), Arizona Fish and Game Department Scientific Collecting permit (#SP708255), Grand Canyon National Park Scientific Research and Collecting permit (#GRCA-2014-SCI-0025), and Colorado State University Animal Care and Use Committee permit (#05-086A-01). All research activities were consistent with American Ornithologists Union guidelines for capturing and handling birds.

### Data analyses

We used 4 metrics to estimate the relative qualities of a disperser’s original and new territory: (1) each territory’s proportional (see below) frequency of occupancy by its ensemble of egg-laying pairs (irrespective of hawk identity); (2) a dispersed hawk’s *z*-scores (see below) of annual fledgling production in its pre-dispersal years compared to its post-dispersal breeding years; (3) the pre-dispersal *z*-score of a dispersed hawk’s annual fledgling production and the *z*-score of fledgling production of the breeder(s) in the disperser’s new territory during those same pre-dispersal years (compares a hawk’s pre-dispersal reproduction in its original territory during the years when a potential disperser could have assessed the reproductive performance of breeders on its future territory); and (4) the *z*-scores of annual fledgling production over all monitored years by the ensembles of breeders in the original and new territory. We also investigated whether divorced hawks that dispersed or stayed improved their reproduction post-divorce by comparing their mean *z*-scores of annual fledgling production before and after divorce. For reasons explained above, all among-territory comparisons of *z*-scores and tests of hypotheses regarding the causes and consequences of breeding dispersal were calculated from the combination of consecutive years of territory and mate retention and the inferred years of retentions in years when resights were missed (the bracketed rule).

Though *z*-scores accounted for variability in the number of years each hawk was monitored as well as annual variation in reproduction due to the variable quality of breeding years, hawks that nested more often had more precise *z*-scores. Based on our earlier findings on the number of locally recruited children and grandchildren in relation to the total number of fledglings a goshawk produced [84] (also see [98]), we assumed a goshawk’s total fledgling production was a reasonable estimate of its future recruitment and therefore its fitness.

To account for variation in years each territory was monitored (11–20 yrs) we calculated a territory’s proportional rate of occupancy by egg-laying pairs by dividing the years it was occupied by egg-laying pairs by the years it was monitored. Because occupancy can increase with territory quality [66], and the discovery of a goshawk territory depends on its occupancy status, we tested for differences in the occupancy rates of territory groups discovered sequentially (36 territories in 1991, 27 new territories in 1992, 18 new territories in 1993, and so on; see S1 Fig in S1 Appendix) with a simple linear regression using ‘group’ as a single categorical explanatory variable and logit-transformed proportional occupancy as the response.

To determine the relative qualities of the territories and mates based on reproduction of hawks before and after they dispersed, we used averaged *z-*scores of fledglings produced,


$${\overset{―}{z}}_{i}=\sum_{y=1}^{n}\frac{{(x}_{iy}-{\overset{―}{x}}_{y})}{s_{y}},$$

where *x*_iy_ denotes the number of fledglings produced by hawk *i* in year *y*, ${\overset{―}{x}}_{y}$ is the mean number of fledglings produced by hawks in year *y*, and $s_{y}$ is the standard deviation of fledglings produced in year *y*. Including the mean number of fledglings produced by hawks across all reproductive territories in year *y* when computing *z*-scores adjusted each hawk’s reproduction in year *y* to account for interannual variation in the quality of a breeding year. For hawks that dispersed multiple times, *z*-scores were separately determined for each territory and mate before and after each dispersal.

We used a binomial generalized additive mixed model (GAMM) to test four leading predictors of breeding dispersal reported for raptors and birds in general: sex, years of familiarity on the original territory, outcome of the prior breeding attempt, and whether the mate was retained at the next breeding attempt [11,15,35,39]. Outcome of the prior breeding attempts by hawks whose identity was known and inferred was treated as a categorical covariate with three levels: (1) failure to produce eggs; (2) failure to produce fledglings given egg laying; and (3) fledglings produced. The GAMM included fixed additive effects for mate retention and outcome of prior breeding attempt, a non-linear interaction using thin plate splines between sex and years of territory familiarity, and territory ID as a random effect to account for repeated measures among territories [99]. Wald tests were used to test the significance for all terms. Because a disperser’s new mate was either unknown (not observed) or seen as unbanded (breeding history unknown) in 10 of the 61 dispersals, the number of dispersals used in the Wald tests was 51. The GAMM model was fit using the *mgcv* package, model estimates were obtained using the *emmeans* package [100], diagnostic tests of model fit were conducted using the *DHARMa* package [101], and graphics were produced using the *ggplot2* package [102]. All analyses were conducted using the statistical software program R [103].

In addition to the GAMM, we investigated the relative importance of a broader suite of individual and environmental variables (inclusive of the GAMM variables) for predicting breeding dispersal with conditional inference forest (CIF) [104]. CIF is a non-parametric random forest method based on conditional inference trees that has high predictive accuracy, can withstand correlated independent variables, can automatically detect higher order interactions without the need to specify them beforehand, is less limited by sample size than traditional parametric methods, and is easy to interpret. Like in the GAMM analysis, we used a reduced data set (due to unknown mate quality metrics of unbanded mates and mates who were never observed) comprised of 26 dispersants whose mates were known (bands resighted) in the CIF model (S2 Table in S2 Appendix). To account for unbalanced response classes in the data (i.e., dispersal much less frequent than territory fidelity), especially among some predictor categories, we applied area under the curve (AUC) as our metric for calculating permuted variable importance [105]. AUC-based variable importance improves the calculation of accuracy for random forests with an “unbalanced” response variable by weighing error rates by predictor category rather than individual observations, which, in turn, improves the detection of changes in tree predictions in less frequently observed predictor categories [106].

All CIF analyses were conducted using the *party* package [107] in R [103]. Explanatory variables and their definitions are listed in Table 1, and all values reported are means ±SE.

**Table 1:** Name and description of environmental and individual hawk variables used in a decision tree classification model, conditional inference forest (CIF), to investigate 21 potential predictors of breeding dispersal in American goshawks on the Kaibab Plateau, Arizona, USA, 1991-2010.

Variable	Description
**Sex**	Male or female goshawk.
**Age**	Hawk age (yrs-old). Fifty-eight male and 88 female breeders were known-aged (recruits banded as nestlings), 4 males and 9 females were aged as 2-year or 3-year olds based on amount of retained juvenal plumage, and 136 males and 153 females were ≥4-year-old based on their full adult plumage at first capture. All hawks aged at ≥4-year-old were assumed to be 4-years-old at that time.
**Mate age**	Age of a focal hawk’s mate (see *Age* above).
**PY Fledge**	Outcome (failure to lay eggs; no young fledged given eggs; fledged ≥1 young) of a hawk’s nest attempt in the year prior to its decision to retain its territory or disperse.
**Eggs**	Did a hawk produce eggs in its prior breeding attempt? True or false.
**Pr Nest Success**	Did a hawk’s prior breeding attempt fledge young? True or false.
**Pr ZProd**	Annually adjusted (standardized) number of young fledged in prior breeding attempt.
**PYrQuality**	An index of the prior year’s quality for breeding based on the proportion of monitored territories with egg-laying goshawks. The proportion of territories monitored in a previous year that had eggs the current year (territory-group based).
**PMateQ1**	Quality of prior (pre-dispersal) mate. Sum of a hawk’s original (pre-dispersal) mate’s annually adjusted *z*-score of lifetime fledgling production.
**PMateQ2**	Quality of prior (pre-dispersal) mate. Average of a hawk’s original (pre-dispersal) mate’s annually adjusted *z*-score of lifetime fledgling production.
**CMateQ1**	Current (post-dispersal) mate quality. Sum of a hawk’s new (post-dispersal) mate’s annually adjusted *z*-score of lifetime fledglings produced.
**CMateQ2** **PTerrQ1**	Current mate quality. Average of a hawk’s new (post-dispersal) mate’s annually adjusted *z*-score of fledglings produced. Prior territory quality. Sum (over all monitored yrs) of annually adjusted *z*-score of total fledglings produced in a disperser’s prior (original) territory.
**PTerrQ2**	Prior territory quality. Average (over all monitored yrs) annually adjusted *z*-score of total fledglings produced in a disperser’s prior (original) territory.
**CTerrQ1**	Quality of current (post-dispersal) territory. Sum (over all monitored yrs) of annually adjusted *z*-score of total fledglings produced in a disperser’s new territory.
**CTerrQ2**	Quality of current territory. Average (over all monitored yrs) of annually adjusted *z*-score of total fledglings produced in a disperser’s new territory.
**YrsOnPriorTerr**	Number of years a hawk was resighted and inferred using the bracketed rule (see Methods) to be in the same territory. A metric of familiarity with a territory.
**BreedExp**	Breeding experience. The number of years a hawk had eggs in the same territory, including the current year.
**Mate BreedExp**	Breeding experience of current mate.
**Pr mate loss**	Is the current mate the same mate as in a hawk’s last breeding attempt? True or false.
**Pr Divorce**	Opposite of mate fidelity. Tallied when a breeding pair separate and both sexes subsequently resighted nesting with new mates.

## Results

### Territory and mate fidelity vs. breeding dispersal

During our 20-year study, we monitored breeding goshawks at 1,688 nest attempts (eggs laid) on 120 territories by 198 color-banded males and 250 color-banded females (differences in counts reflect the relatively difficulty of trapping/banding males vs. females). All territories that lost or received a disperser fell within a subsample of territories numbered 1–116, all of which were monitored 11–20 years (S1 Table in S2 Appendix, S1 Fig in S1 Appendix). We therefore used the territories 1 –116 to investigate goshawk fidelity to territory and mate and the correlates and consequences of breeding dispersal. The identities (bands resighted) of 264 breeding goshawks (102 males; 162 females) in the 1–116 territories were known in ≥2 years and were therefore available for estimating fidelity to territory and mate. All banded and breeding hawks were recorded at each year’s resights as ‘retained its territory,’ ‘retained its mate,’ ‘dispersed to a new territory and new mate,’ or ‘dispersed with its mate to a new territory.’

The annual proportions of the territories occupied by egg-laying pairs was highly variable (see Background and field methods; S1 Table in S2 Appendix), much of which was due to 35% of females (56 of 162 females whose identities were known in ≥2 yrs) who temporally skipped breeding (along with their males) in a total of 77 years, 48% of which occurred in 1 (1994, 1997) to 3 successive poor breeding years (2001–2003, 2007–2009; S1 Table in S2 Appendix). Eight other hawks skipped breeding in a total of 13 years, all between apparent mate loss and pairing with a new mate. Despite extensive annual variation there were no significant overall differences (*p* = 0.59) in the proportions of years each of the 11 groups of territories were occupied by egg-laying pairs (S2 Fig in S2 Appendix). This suggests that variation in a year’s quality for nesting similarly affected each group irrespective of the year territories were discovered.

Our monitoring identified 61 breeding dispersals (17 by males, 44 by females) and showed that 56 (48%) of the 116 territories either lost or received a disperser. The majority (79%; 48 of 61) of dispersals comprised a once in a lifetime movement by breeders. However, 5 hawks (1 male, 4 females) dispersed twice and 1 female dispersed 3 times. Three of the 5 that dispersed twice moved back to their original territory in their second move 3–6 years later. Of 49 territories from which a hawk dispersed, 39 (80%) had a single departure, 8 (16%) had 2 departures, and 2 (4%) had 3 departures. Of the 43 territories that received a dispersed hawk, 31 (72%) received 1 disperser, 7 (16%) received 2, 4 (9%) received 3, and 1 received 4 (2%). Twenty-six (43%) of the 61 dispersals occurred in consecutive years showing that the dispersal process can occur from 1 year to the next. In contrast, 35 (57%) of the 61 dispersed hawks were not seen in their old or new territory for 2–5 years ($\overset{―}{x}$ = 2.1 ± 0.16, median = 2), indicating the dispersal process can take multiple years. It was not known if the dispersers that took >1 year to disperse joined a floater pool before settling in their new territories.

When computation of breeding dispersal rates was limited to consecutive-year territory retentions (no bracketed inferences), there were 212 male territory retentions and 17 within study area dispersals totaling to 229 opportunities to detect dispersal giving a male breeding dispersal rate of 0.07 (17/229) and a territory fidelity rate of 0.93. For females, there were 333 consecutive-year territory retentions and 44 dispersals totaling to 377 opportunities giving a dispersal rate of 0.12 (44/377) and a territory fidelity rate of 0.88. When computation of dispersal rates for 48 males included the 212 consecutive year resights plus 105 years of inferred territory retentions with the bracketed rule (72 1-yr retentions, 26 2-yr retentions, 6 3-yr, and 1 4-yr), the 317 total retentions yielded a male dispersal rate of 0.05 (17/334) and a territory fidelity rate of 0.95. For 69 females, the bracketed rule added 120 inferred retentions (93 1-yr inferred retentions, 18 2-yr, 6 3-yr, 2 4-yr, and 1 5-yr) to the 333 consecutive-year retentions resulting in a female dispersal rate of 0.12 (44/497) and a territory fidelity rate of 0.88.

Combining the consecutive years of territory retention with the inferred years of retention of their original territory prior to dispersal, the years of territory familiarity ranged from 1–7 years, breeding experience ranged from 2–6 years, and the range in age of both sexes was 3–13 years (Table 2, S3 and S4 Figs in S2 Appendix). Mean age at dispersal of males was ~ 1 year older than females, males had slightly more familiarity and breeding experience on their original territory than dispersed females, and there was little difference in the means and ranges of the ages of the original and the new mates for both sexes.

**Table 2:** Means ±SE, medians, and ranges at the time of dispersal of male and female ages (yr-old), territory familiarity (yr territory occupied), breeding experience (number of nest attempts), and the breeding experience and ages of new mates on their post-dispersal territories of 17 male and 44 female American goshawks that changed territories on the Kaibab Plateau, Arizona, USA, 1991-2010.

	Old territory		New territory	
	male	female	male	female
**Age**^a^ **(yrs)**	7.8 ± 0.65 7 (4‒13)	6.9 ± 0.3 6.5 (3‒11)	–	–
**Territory familiarity (yrs)**	2.4 ± 0.46 2 (1‒7)	1.7 ± 0.19 1 (1‒6)	–	–
**Breeding experience (yrs eggs laid)**	2.2 ± 0.39 2 (1‒6)	1.8 ± 0.16 1 (1‒5)	–	–
**Mate age**^**a**^ **(yrs)**	5.8 ± 0.71 5 (2‒11)	5.6 ± 0.36 5 (2‒10)	6.1 ± 0.6 6 (2‒10)	5.9 ± 0.51 5 (2‒11)

^a^All hawks aged at first capture as ≥4-years-old (based on full adult plumage) were assumed to be 4-year-old (see Methods).

#### Mate fidelity vs. divorce.

Near lifetime mate fidelity characterized the pair bond in Kaibab goshawks. Pair duration ($\overset{―}{x}$ = 1.7 ± 0.07, median = 1.0, range = 1–7 yr) was determined from 442 pairings of banded hawks of which 91 pairs nested in multiple years (170 pairs were observed to have nested only once) totaling to 261 years of mate retentions. Ten divorces were detected with only one hawk, a male, divorcing twice. There was some evidence that females, more often than males, initiated divorce. In 7 of the divorces the female dispersed while their males stayed on their territories, whereas in 2 cases the male dispersed while the females stayed, and in two cases both pair members dispersed post-divorce. In one of the latter two cases, we could not tell which mate left first, but the female first nested with a new mate 2 years post-divorce, while the male was observed nesting in his new territory 3 years post-divorce. Of the 2 males that stayed post-divorce, 1 nested with a new female on his original territory for 2 years before he dispersed on loss of that female to nest again with his original female on a territory adjacent to their original. The other male stayed on its territory to nest with a new female but he then dispersed after loss (never observed again) of this second mate. Given the two of 10 divorces in which both partners dispersed, 12 (20%) of the 61 observed dispersals were the consequence of divorce.

On computation of the divorce rate using 181 total consecutive-year mate retentions, the divorce rate was 0.06 (11/181) with a mate fidelity rate of 0.94. When the divorce rate included consecutive year mate retentions plus years of inferred mate retentions, the bracketed rule added 22 inferred retentions (15 1-yr inferences, 2 2-yr inferences, and 1 3-yr inferences) to the 181 consecutive year retentions, resulting in a divorce rate of 0.05 (10/203) and a mate fidelity rate of 0.95, a difference of only about 0.01 between these methods for estimating the divorce rate.

### Dispersal distances

**Fig 2:**
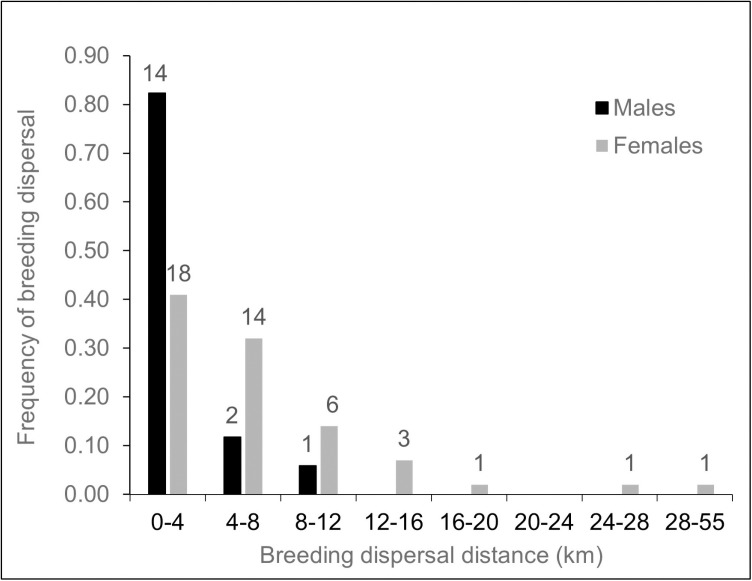
Frequency and distance of breeding dispersals made by male and female goshawks in Arizona, USA. All male and 62% of female dispersals moved no further than to a 3rd-order neighboring territory (i.e., crossed two territories); maximum female dispersal was 51.2 km on the Kaibab Plateau, Arizona, USA, 1991-2010.

Mean nest-to-nest dispersal distance for the 17 males was 3.3 ± 0.6 km (median = 2.3, range, 1.8 –11.2 km) and all dispersals were to territories within the male’s 3^rd^ order neighborhood of territories (i.e., crossed 2 territories; Fig 2). Mean nest-to-nest dispersal distance for the 44 females was 7.5 ± 1.3 km (median = 4.6, range, 1.9–51.2 km), significantly farther than males (*t* = 3.3, df = 14.6, p = 0.005). Though the longest female dispersal crossed 13 territories, 38 (86%) of dispersed females also moved to territories no farther then to their 3^rd^ order neighborhood. Mean centroid-to-centroid dispersal distances (male, 3.3 km; female, 7.5 km) were nearly identical to the nest-to-nest distances, a consequence of the fact that dispersed hawks typically nested in or close to the same nests used by previous territory owners.

Mean dispersal distance (nest to nest) by divorced males (3.4 ± 0.7, range = 2.3–6.2 km, *n* = 5) was essentially the same as the dispersal distances by non-divorce males (3.3 ± 0.8 km, range = 1.8–11.2 km, *n* = 12), whereas mean dispersal distance of divorced females (4.2 ± 1.1 km, range = 1.9–11.0 km, *n* = 8) was half the dispersal distance of non-divorced females that dispersed (8.3 ± 1.5 km, range = 1.9–51.2 km, *n* = 36), reflecting the 3 dispersals >16 km by larger sample of non-divorced females.

### Predictors of dispersal

Our GAMM, which included mate retention or loss, the prior nest attempt outcome (no eggs laid; eggs, but nest failed; fledglings produced), and a non-linear interaction between sex and years of familiarity of the original territory, had a classification accuracy of 0.94. A within-sample Kappa statistic (an out-of-sample goodness of fit model did not converge due to small sample size) showed that this classification was only fair (k = 0.4). This was not surprising given that only 61 hawks dispersed out of 831 opportunities to detect dispersal; there was a good chance of correctly guessing that a hawk ‘did not disperse’.

**Fig 3:**
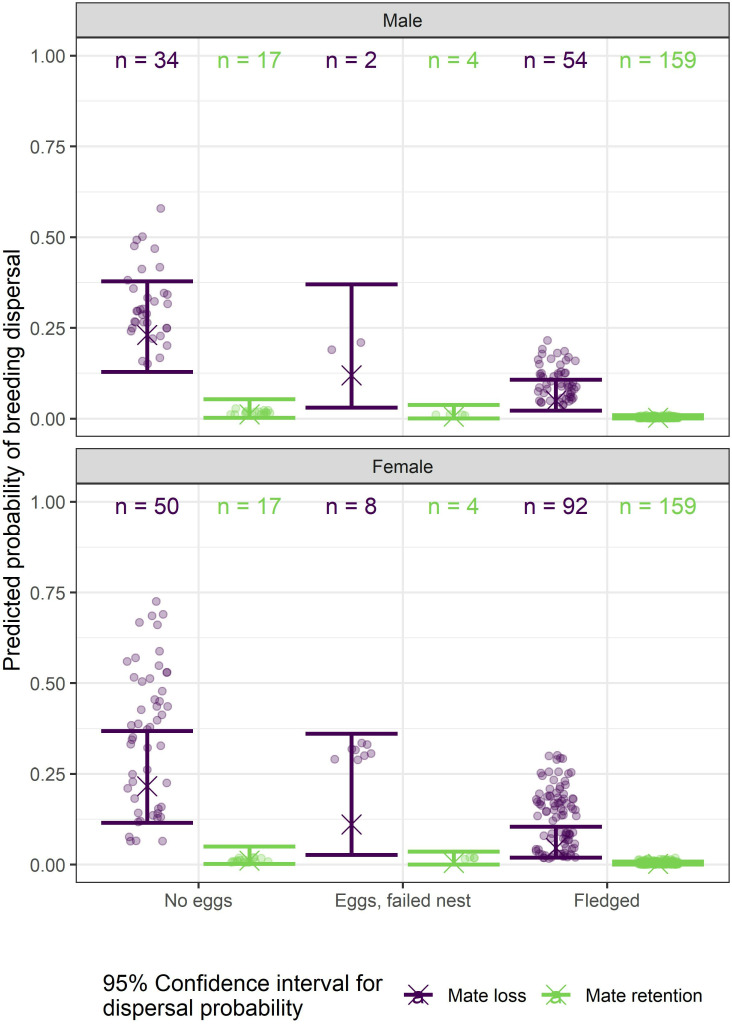
Probability of goshawk breeding dispersal given sex, prior nesting outcome, and mate retention in Arizona, USA. Dispersal probabilities with 95% confidence intervals (bracketed lines) within combinations of sex, outcome of the prior nest attempt (no eggs laid; eggs laid but nest failed; fledgling produced), and mate retention or loss (retention in green and loss in purple) by goshawks on the Kaibab Plateau, Arizona, USA, 1991-2010. Points show individual predictions (fitted values) while predicted means are denoted by “X.”.

**Fig 4:**
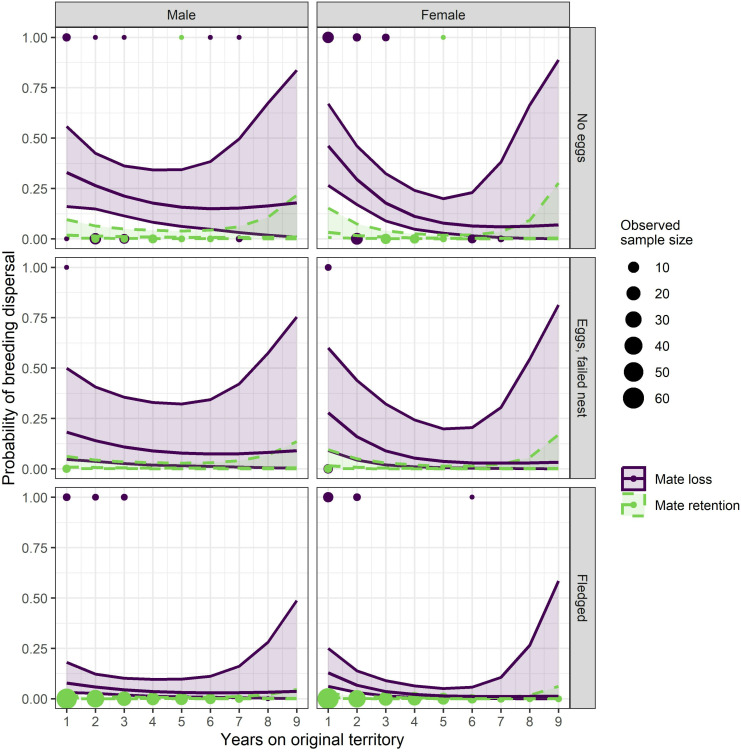
Probability of goshawk breeding dispersal given sex, territory familiarity, prior nesting outcome, and mate retention. Means and 95% confidence bands of predicted probabilities of breeding dispersal across years of a goshawk’s familiarity with its territory given combinations of sex, outcome of the prior nest attempt (no eggs laid; eggs laid but nest failed; fledgling produced), and mate retention (dashed line) or loss (solid line) on the Kaibab Plateau, Arizona, USA, 1991-2010. Sample sizes of dispersals (top line) and non-dispersals (bottom line) are depicted by point size and colored by mate retention or mate loss.

Holding the years of familiarity with the prior territory constant at a mean of 2.6 years and averaging over sex, the GAMM model showed that the highest predicted probability of dispersal (0.22) occurred when (1) a hawk’s mate was not retained (mate loss) at the next breeding attempt and the pair failed to lay eggs in the previous breeding attempt, (2) dispersal declined marginally (0.11) when a pair’s prior nest attempt failed after laying eggs and the mate was not retained, and (3) declined further (0.05) when a pair’s prior nest attempt fledged young and the mate was lost (Table 3, Fig 3, S3 and S4 Tables in S2 Appendix). In contrast, dispersal probability was lowest (0.002) when (1) a hawk’s mate was retained and the pair successfully fledged young in their prior nest attempt, (2) the mate was retained after the pair’s prior nest failed after laying eggs in the prior attempt (0.005), or (3) the mate was retained but the pair failed to lay eggs in the prior attempt (0.01). The predicted probability of dispersal with any number of years of territory familiarity was low when the pair successfully fledged young in the prior attempt. The non-linear interaction between years of territory familiarity and sex showed the probability of dispersal decreasing with years of familiarity before increasing slightly when either member of a pair had more than seven years of territory familiarity. The initial decrease in probability of dispersal was most prominent in females (Fig 4).

**Table 3:** Probability of goshawk breeding dispersal given the prior nesting outcome and mate retention in Arizona, USA. Predicted probabilities of breeding dispersal (sexes combined) following outcome of the prior nest attempt (no eggs laid; eggs laid, nest failed; fledglings produced), mate retention or loss at the next nest attempt, and averaging across sex and years of territory familiarity of American goshawks on the Kaibab Plateau, Arizona, USA, 1991-2010.

Nest	Mate Retained	Probability of dispersal
**No eggs laid**	yes	0.0112
**Eggs, nest failed**	yes	0.0051
**Fledglings produced**	yes	0.0020
**No eggs laid**	no	0.2231
**Eggs, nest failed**	no	0.1148
**Fledglings produced**	no	0.0474

There was weak evidence that low numbers of fledglings produced in the prior breeding attempt influenced the probability of dispersal; the mean annual *z*-score of fledged young in the prior nest attempt by the 61 dispersed hawks was below average (-0.02 ± 0.13) whereas the mean annual *z*-score of fledged young in the prior attempt that did not disperse was only slightly above average (0.14 ± 0.03 fledglings). Lastly, we were unable to detect individual hawk differences in the propensity to disperse multiple times because of the 55 hawks that dispersed, only 6 (11%) did so multiple times. Excepting the 12 divorce dispersals, two pairs that dispersed together, and 10 cases where a mate was unbanded, 37 of a disperser’s lost mates were never observed again after their last breeding attempt, suggesting their death or possible emigration from the Kaibab Plateau.

**Fig 5:**
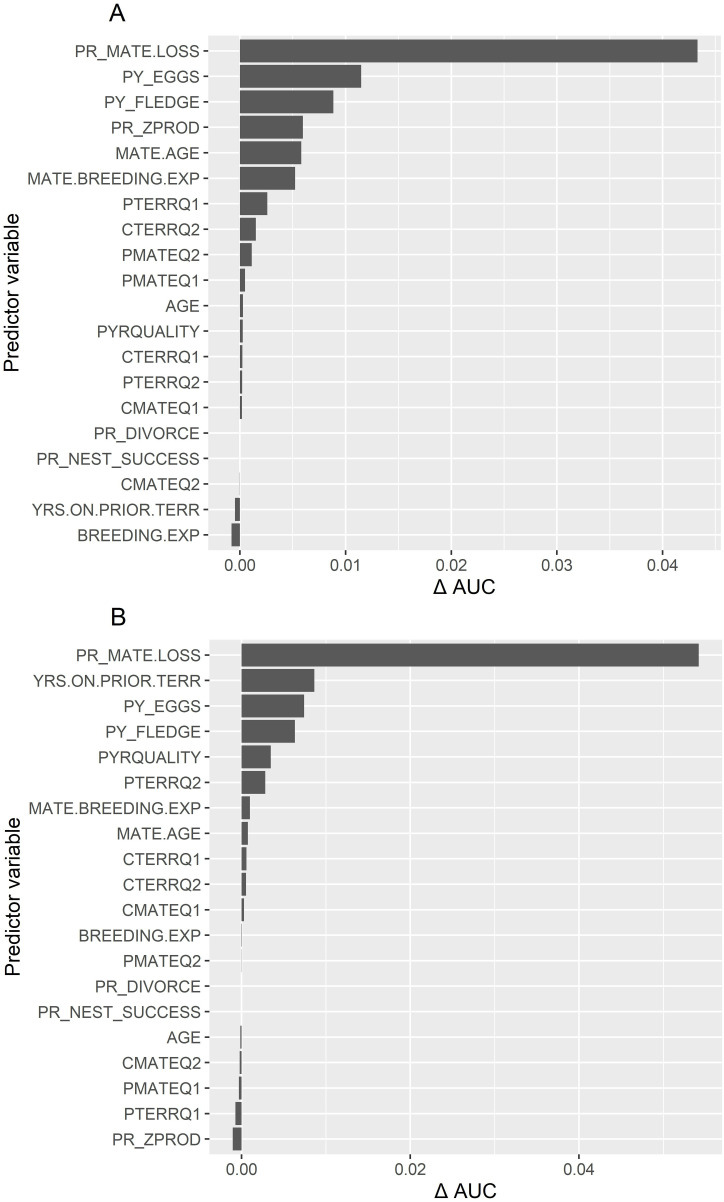
Conditional Inference Forest importance of 21 variables for predicting goshawk breeding dispersal in Arizona, USA. Permutation-based area under the curve (AUC) from conditional inference forest (CIF) of the importance of 21 individual and environmental variables for predicting breeding dispersal by 13 male (A) and 13 female (B) American goshawks on the Kaibab Plateau, Arizona, USA, 1991-2010. Length of bars show the relative importance of each variable measured by the difference in predictive accuracy of the model using the permuted values versus the original values. Variable descriptions are in Table 1.

The CIFs for both sexes confirmed that mate loss (*PR_MATE.LOSS*) was by far the strongest predictor of dispersal (Fig 5). The importance of mate loss on a male’s decision to disperse was distantly followed by whether the pair had produced eggs in the prior nest attempt (*PY_EGGS*) and whether the previous year’s nest attempt was successful (*PY_FLEDGE*). The importance of mate loss on a female’s dispersal was distantly followed by years of territory familiarity on her original territory (*YRS.ON.PRIOR.TERR*), and whether they produced eggs in the prior attempt (*PY.EGGS*). Interestingly, years of territory familiarity was of little importance to males, as was divorce (*PR.DIVORCE*) as a predictor for both sexes, likely due to its rarity.

### Consequences of dispersal

**Fig 6:**
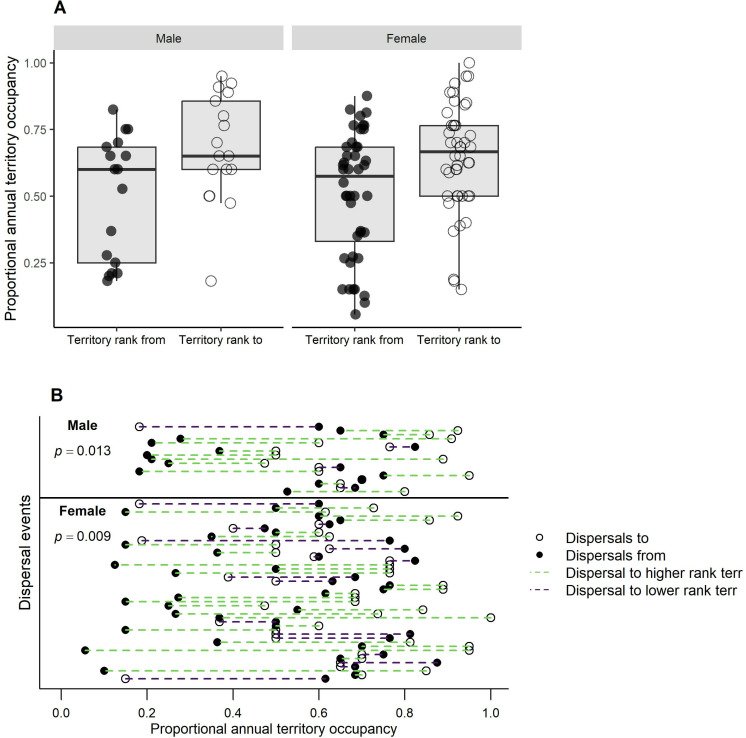
Did dispersing goshawks move to more frequently occupied territories in Arizona, USA? (A) Box plots of the proportional years of territory occupancy (yrs occupied/yrs territory monitored) by egg-laying pairs in territories from which hawks (*n* = 61) dispersed from vs. to. (B) Display of changes in the total (all monitored yrs) proportional years of occupancy of an original vs. new territory by individual dispersal events on the Kaibab Plateau, Arizona, USA, 1991-2010.

**Fig 7:**
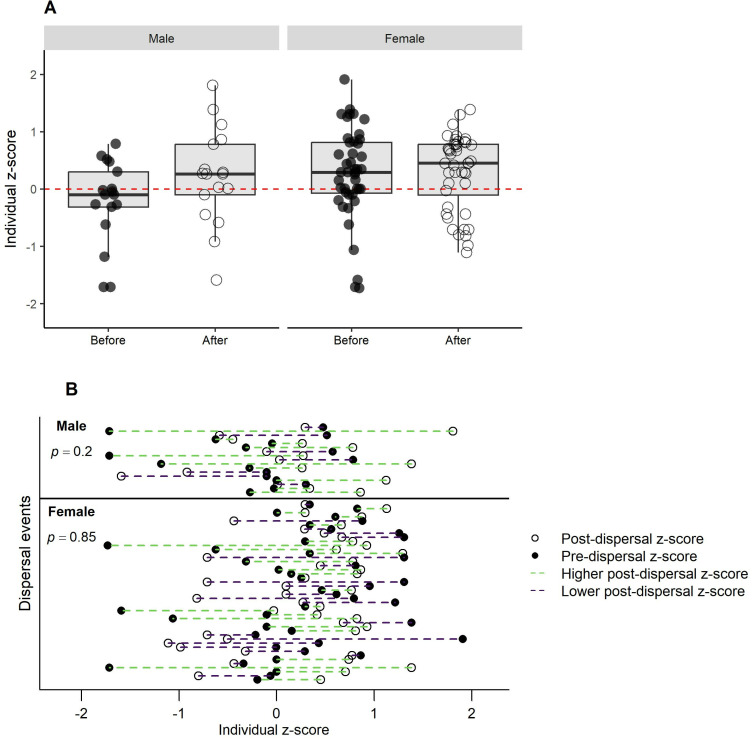
Did dispersed goshawks improve their post-dispersal reproduction in their new territories in Arizona, USA? (A) Box plots of mean annual *z*-scores of fledgling production by dispersed hawks in their original territories vs. their new territories. (B) Display of changes in mean annual *z*-scores of fledgling production in an original vs. new territory by individual dispersal events on the Kaibab Plateau, Arizona, USA, 1991-2010.

**Fig 8:**
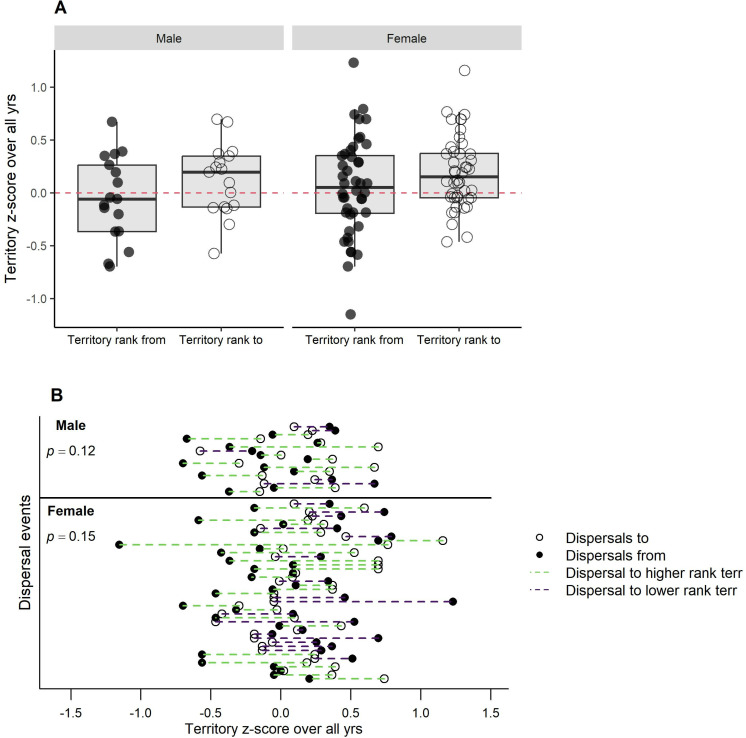
Did dispersed goshawks settle in territories with greater (all monitored years) reproduction in Arizona, USA? (A) Box plots of differences in the mean *z*-scores of annual fledgling production by the complete (all monitored yrs) ensemble of breeders in territories which male and female goshawks dispersed from and to. Red horizontal line indicates equal quality rankings (*z*-scores) of original and new territory. Positive values show higher productivity in the new vs. old territory and negative values show lower productivity in the new territory. (B) Display of changes in mean *z*-scores of annual fledgling production by individual goshawks that dispersed to new territories on the Kaibab Plateau, Arizona, USA, 1991-2010.

**Fig 9:**
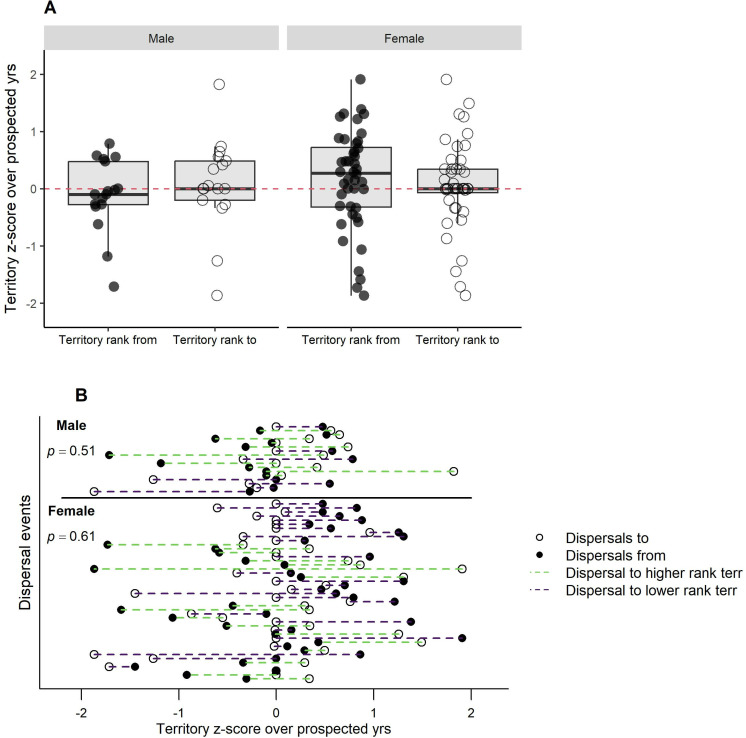
Did dispersed goshawks settle in territories with hawks that out-produced them during the disperser’s pre-dispersal years? Fledgling production by dispersers in their original territories during the years when they could via territory intrusions monitor the nesting success in neighboring territories prior to their dispersal. (A) Differences (new ‒ old territory) in mean annual *z*-scores of fledgling production in an original vs. new territory where *z*-scores were averaged across those years when a prospective disperser could have monitored the reproduction of a breeder(s) in the new territory prior to their dispersal. Red horizontal line indicates no difference (i.e., equal quality rankings of original and new territory or mate), positive values show higher productivity and negative values depict lower productivity. (B) Display of differences in mean annual territory *z*-scores of fledgling production for individual dispersal events across prospecting years in original territories (closed black circles) and new territories (open circles).

On average, dispersals by males and females were to territories that were significantly more often occupied by egg-laying breeders (males, *t*_16_ = -2.81, p = 0.013, females, *t*_43 _= -2.74, p < 0.009) (Fig 6). There were, however, no significant changes in *z*-scores of annual fledglings produced by dispersed males (*t*_16_ = 1.35, p = 0.196) or females (*t*_43_ = 0.19, p = 0.852) in *t*heir original vs. their new territories (Fig 7). There were also no significant differences in the total (all monitored yrs) territory mean *z*-scores of fledglings in their original and new territories (males, *t*_16_ = -1.66, p = 0.117; females, *t*_43_ = -1.48, p = 0.146) (Fig 8), and *t*here were no significant differences (males, *t*_16_ = -0.67, p = 0.510; females, *t*_43_ = 0.51, p = 0.609) in a hawk’s pre-dispersal *z*-scores of annual reproduc*t*ion and the *z*-scores of breeder(s) in the disperser’s new territory during the same pre-dispersal years (Fig 9).

#### Divorce and its consequences.

Nest failure was a poor predictor of divorce as 7 of the 10 divorces occurred after successfully fledging young; only 3 divorces were preceded by nest failure after producing a clutch. Divorce did not improve the fitness of hawks as there were no significant changes in *z*-scores of annual fledglings produced before and after divorce by hawks that changed territories or hawks that stayed on their original (males, *t*_*9*_ = -0.76, p = 0.467; females, *t*_*9*_ = 0.05, p* *= 0.963) (see S5 Fig in S2 Appendix). For both sexes, age, breeding experience, and years of familiarity in their original territory at divorce appeared to have little effect on the decision to divorce as the ranges in age, experience, and territory familiarity encompassed the full range of these variables observed in the population of divorced and non-divorced breeders (Table 4). Nonetheless, at the time of divorce, the means of age, years of breeding experience, and territory familiarity of divorced and dispersed males were 1–2 years greater than the males that stayed; a difference not seen in divorced females whose ages, familiarities, and experiences were essentially the same among divorcees that dispersed vs. stayed. There was circumstantial evidence of forced divorce via intra-sex competition (see [54]) as 2 female and 3 male divorcees that dispersed were each replaced by new mates in the next breeding season.

**Table 4:** Ages, territory familiarity and breeding experience, and ages of new mates of goshawks that dispersed or stayed. Mean ±SE and median and range in the second row of male and female ages (yr-old), years of territory familiarity (first to last breeding), years of breeding experience (years of egg-laying) on original (pre-dispersal) territory, and ages of original and new mates of males and females at the time of divorce for divorced (*n* = 10 divorces) American Goshawks that stayed or dispersed to new territories on the Kaibab Plateau, Arizona, USA, 1991- 2010.

	Stayed		Dispersed	
	Male	Female	Male	Female
**Age** ^a^	5.0 ± 0.25 5 (4‒7)	5.3 ± 0.77 4 (4‒8)	7.2 ± 0.48 7 (4-10)	6.0 ± 0.34 5 (4‒10)
**Territory familiarity**	2.0 ± 0.45 1 (1‒6)	2.3 ± 0.77 1 (1‒5)	4.0 ± 0.49 3 (1‒7)	2.3 ± 0.32 1 (1‒6)
**Breeding experience**	1.4 ± 0.12 1 (1‒3)	2.0 ± 0.58 1 (1‒4)	3.2 ± 0.39 3 (1‒6)	2.1 ± 0.21 2 (1‒5)
**Age**^**a**^ **of original mate at divorce**	6.0 ± 0.51 5 (4‒10) (*n *= 5)	5.7 ± 0.51 6 (4‒7) (*n *= 3)	5.6 ± 0.44 4 (4‒8) (*n *= 5)	6.3 ± 0.35 5 (4‒10) (*n *= 7)

^a^All hawks aged at first capture as ≥4-years-old based on full adult plumage were assumed considered to be 4-years of age (see Methods).

## Discussion

Using a long-term data set on a large population of color-banded goshawks that nested in ≥2 years (opportunities to detect dispersal and divorce) whose identities were known (band resighted), or inferred with the bracketed rule (see Methods), totaling to 831 (334 male and 497 female) opportunities to detect breeding dispersal, we showed that breeding goshawks on the Kaibab had strong territory and mate fidelities; 92% of males and 84% of females showed lifetime territory fidelity and 95% of hawks showed lifetime fidelity to their mate. However, due to the commonness of hawks skipping breeding years and the uncertainties associated with our frequent application of our bracketed rule (225 times for 117 banded hawks) due to the near zero detection rate of non-breeders, we reported two rates of territory fidelity and of mate fidelity. First, we reported rates based on years of a hawk’s retention of its territory and mate only when the retentions detected in consecutive breeding years on the same territory. Second, we reported retention rates based on the years of a hawk’s consecutive years plus the years when our bracketed rule inferred territory and mate retention during skipped years when resighted on the same territory. Given the frequency with which goshawks skipped egg-laying combined with the evidence validating our bracketed rule, we contend that inclusion of the inferred years of a hawk’s retention of its territory and mate resulted in more accurate estimates of their true territory and mate fidelities.

Nonetheless, we recognize that our fidelity estimates may be biased high because breeding hawks that remained year-to-year in the same territory are more likely to be resighted than dispersed individuals [91,92]. Additionally, some dispersals may have been missed (1) when their nests failed before they could be resighted, (2) if unbanded breeder that eventually dispersed was incorrectly tallied as a new recruit, (3) if a “disperser” joined a non-breeding floater pool, and (4) if a disperser emigrated from the Kaibab Plateau. Except for possible emigration, any bias due to the first three contingencies should have declined substantially after our third study year (1993) when the great majority of the potential territories on the Kaibab Plateau were under intensive monitoring. While the frequency of emigration from the Kaibab Plateau was unknown, suggestions of its uncommonness include the maximum observed dispersal of 51 km in our study area where movements of up to 78 km could have been detected. Furthermore, of 6 concurrent goshawk nest monitoring studies within 400 km of the Kaibab, 2 reported natal dispersals (natal to first breeding site) from the Kaibab (1 to the GCNP-South Rim at 55 km, T. S. Bowden, personal communication; 1 to Utah’s Dixie National Forest at 107 km, D. J. Worthington, personal communication), whereas none reported breeding dispersals (Arizona’s Apache-Sitgreaves National Forest, M. F. Ingraldi, personal communication; Arizona’s Coconino National Forest, P. Beier and T. Drennan, personal communications; and 6 Utah National Forests, S. A. Sonsthagen and J. Underwood, personal communications). Despite the above potential sources of bias, we are confident that our 20-year findings on the large sample of banded breeders in an insular forested area increased our understanding of the causes of dispersal and divorce, dispersal distances, and the consequences of dispersal by American goshawks.

### Predictors of dispersal

Our GAMM investigation of the correlates of breeding dispersal, which included 51 of the disperser’s new mate’s breeding history was known (see Methods), showed that mate loss (i.e., via dispersal, emigration, or mortality) was the best predictor of breeding dispersal; 96% of the 51 dispersals in our GAMM investigation followed mate loss (two pairs dispersed together; no mate loss). Averaging across all other dispersal predictors, mate loss alone had a predicted probability of dispersal of 0.11 compared to 0.005 for hawks that retained their mate. The lowest probability of dispersal (0.002) occurred when a pair’s previous nest attempt produced fledglings and the mate was retained at the next breeding attempt. Failure of a pair’s prior nest attempt (eggs laid) had a minor effect on the probability of dispersal (averaging across all other variables, the probability of dispersal for no eggs was 0.05, 0.03 for eggs laid but nest failed, and 0.01 for successful fledging) as only 3 (6%) of the 51 GAMM dispersals followed nest failure when the mate was retained. Ten (16%) of the 61 total dispersals were made by departing divorcees, but divorce, likely because there were so few, was a poor predictor in the CIFs. Despite the importance of mate loss to dispersal, the great majority of both sexes who lost their mate waited on their original territory for a new mate and some long-lived hawks nested in their original territory with up to 5 different sequential mates [83].

Our CIF analyses of a broader suite of individual and environmental predictors confirmed the preeminence of mate loss as a predictor of breeding dispersal. The CIFs of both sexes also showed that previous nest outcomes (whether eggs were laid in the prior year and whether the previous nest was successful) were marginally important, a finding in accordance with many bird studies, including raptors, where prior nest failures were strong predictors of dispersal [17,23,49,50,56,108–110]. Finally, the CIF analyses showed that age in both sexes was a poor predictor of dispersal on the Kaibab.

### Consequences of dispersal

Despite evidence of movements to territories significantly more often occupied by egg-laying breeders and the more frequent changes of territories by breeders following 1 year of territory occupancy typically younger hawks, there was otherwise little evidence that dispersed goshawks on average followed the IDD model of territory selection by moving to higher quality territories. On average, dispersed hawks did not improve their post-dispersal annual reproduction, nor did they move to territories that had higher, long-term, reproduction. Similarly, divorce followed by dispersal did not improve the quality of the divorcees’ new territory or mate as judged by pre- vs. post-divorce dispersal reproduction. Interestingly, breeding female Eurasian goshawks (*A. gentilis*) in Finland dispersed to what was thought to be higher quality territories (from territories with more peatland to those with more forest) but showed no subsequent effects on reproductive performance [22].

### Territory and mate fidelity

Territory fidelity by goshawks on the Kaibab Plateau was in the upper range recorded for the American goshawk. In 9 years of monitoring 28 territories in California, Detrich and Woodbridge [111] reported a male fidelity of 0.76 (4 dispersals, 17 opportunities to detect dispersal) and a female fidelity of 0.79 (14 dispersals, 66 opportunities). Bechard et al. [112] reported a male territory fidelity of 0.96 (1 dispersal, 27 opportunities) and a female fidelity of 0.87 (8 dispersals, 62 opportunities) in 12 years while monitoring 41 territories in Nevada and Idaho. A study of 28 goshawk territories in Southeast Alaska reported a territory fidelity of 1.00 for males (28 males, no dispersals) and, though a female fidelity rate was not reported, 13 breeding dispersals (movements >3.2 km) were made by 11 females [113]. Among Eurasian goshawks, Tolvanen et al. [92] reported territory fidelity in two study areas: 0.92 for 22 males and 0.62 for 13 females monitored 17 years; and 0.97 for 29 males and 0.94 for 16 females monitored 15 years. In another Finnish study, Otterbeck et al. [22] reported that 16 of 55 (29%) female goshawks changed territories over 17 years.

### Dispersal distance

Dispersal frequency and distances moved appear to be contingent on the distribution and density of territories, which can vary by geographically as well as by habitat [13,112,114,115]. Territories on the Kaibab were spaced at a mean 3.8 km [89] and most breeding dispersals were short and to territories within the disperser’s 3^rd^-order pre-dispersal neighborhood. With the exception of a wider range of dispersal distances by females, the distances on the Kaibab ($males,\;\overset{―}{x\;}$ = 3.3, range, 1.8–11.2 km, *n* = 17 dispersals; females, $\overset{―}{x\;}=\;$7.5 km, range, 1.9–51.2 km, *n* = 44 dispersals) were similar to goshawk dispersals in Northern California (males, $\overset{―}{x\;}$ = 6.5, range = 4.2–10.3 km, *n* = 3 dispersals; females, $\overset{―}{x\;}$ = 9.8, range = 5.5–12.9 km, *n* = 4 dispersals [111]; for 7 females in Nevada ($\overset{―}{x\;}$ = 5.8, range = 1.3–10.6 km, n = 8 dispersals) and a single dispersed male (2.1 km) [112]. Both the mean and range of dispersal distances by 6 female Euroasian goshawks in Norway ($\overset{―}{x\;}=10.2$, range = 2.5–42 km, *n* = 8 dispersals [92] were similar to Kaibab goshawks. The maximum female dispersal in our study (51.2 km) was longer by a factor of 4 than for females in California and Nevada, a difference likely related to our relatively large study area. However, the maximum female breeding dispersal in Southeast Alaska was longer (152 km) than reported in any study; discovery of this dispersal was likely the consequence of using telemetry in the naturally fragmented forests in Alaska’s Southeastern island archipelago [113].

More frequent dispersal (not significant in this study) and significantly longer movements by females than males are typical of raptors [11,13,24,37,46,114–120]. Sex roles of raptors differ during breeding. These differences likely reflect the need for males to compete with others to gain and defend territories, court females, and provision their mates and nestlings with food through the nesting period, while females incubate, brood, and defend nest contents until late in the nestling stage [121]. A male’s energy expenditure in establishing and defending a territorty combined with his experience with resource locations in its home range likely lowers a male’s propensity to change territories. Also, shorter male dispersals could reflect smaller home ranges than those of females. Smaller ranges would limit males from prospecting as many distant territories as females whose home ranges can expand via widening excursive movements as the breeding season progresses [93]. One advantage of short dispersals is that the disperser remains close to its familiar territory to which it can return if widowed again.

### Divorce and dispersal

Divorce rates in monogamous birds vary widely within and among species and it is generally thought that divorce is an adaptive strategy whereby individuals maximize their fitness by dispersing to a better territory or mate (the better option hypothesis; [32,39]). Divorce on the Kaibab was uncommon and there was little evidence as to its cause and which sex initiated it. Breeding failure, a common predictor of divorce in birds [32], was a poor predictor of divorce among Kaibab goshawks as seven divorces followed successful fledging of young while only 3 were preceded by failure to fledge after producing a clutch. Other possible causes include ejection of a hawk in intra-sex competition for a territory (the forced divorced hypothesis; [32,54], and accidental separation due to extreme weather or injury preceding or during courtship (the accidental loss hypothesis; [32]). Possible competitive ejection was suggested by 4 females and 2 males that divorced but stayed on their original territory where they nested with a new mate the next breeding season, and an accidental separation was suggested by the repairing of a divorced pair 3 years later in a territory adjacent to their original. Like non-divorce dispersals, divorced and dispersed goshawks moved to nearby territories whose qualities based on reproduction were not significantly different. Goshawks are relatively long-lived and their strong fidelity to both territory and mate make it difficult to distinguish territory fidelity from mate fidelity. Despite this, the two pairs that dispersed together and the post-divorce re-pairing in a territory different from their original, showed that mate fidelity can occur independently of territory fidelity.

### Synthesis: territory choice and dispersal distance

Many widowed or divorced goshawks stayed on their territories for ≥1 years. Options were to wait for a new mate and risk missing a breeding season or leaving their territories to prospect for unoccupied territories or unpaired mates and possibly losing their familiar territory. Our findings suggest that to avoid missing a breeding season and losing a territory, widowed goshawks used a home-based mate searching strategy that included waiting for a new mate while making short-distance prospecting intrusions into nearby territories for unpaired mates (see also [73,75,93]). The apparent non-ideal choice of territories by dispersed goshawks appeared to be a combination of their spatially limited prospecting, the limited sample of closely surrounding territories, and the fortuitous availability of unpaired mates. These contingencies appeared to result in random territory choices with a near equal numbers of hawks moving to higher or lower quality territories. While we were unable to always confirm the presence of an unpaired mate in a disperser’s new territory, the actual occupancy of future territory by an unpaired mate (a singleton) in 29 (48%) of the 61 dispersal territories is suggested by the disappearance (never seen again) of the singleton’s prior mate after their last nest attempt prior to the dispersal. In 32 (52%) of the remaining dispersals, we could not determine which member of an eventual new pair arrived first on their new territory because 22 of the dispersers paired with new recruits, 2 pairs dispersed together, and a single divorced pair re-paired in a territory different than their original.

As predicted by the IDD model that breeding dispersal should more often occur in young breeders (those with little experience on a territory), the probability of dispersal by Kaibab goshawks was greater for individuals following their first year of territory occupancy than breeders with ≥2 years of territory experience (i.e., older hawks). However, the proportion of hawks that dispersed following one year of experience (0.16, *n* = 29 dispersals) was just twice a constant proportion (0.07–0.08, *n* = 22 dispersals) of hawks with 2–7 years (the maximum yrs of experience) of a disperser’s experience on a territory. Thus, while territory change was more frequent following a hawk’s first breeding attempt, dispersal also occurred in older hawks. Older goshawk pairs are more likely to lose a mate through death and older widowed hawks often waited for a new mate for multiple years before finally giving up and dispersing. Moreover, both goshawk sexes that divorced often dispersed at advanced ages. The wide ranges of a disperser’s age, years of breeding experience, and years of territory familiarity, combined with our findings that dispersed hawks did not on average improve their reproduction by moving to better territories, showed that the IDD model can be a poor predictor of dispersal. Alternatively, the apparent non-ideal choice of territory may reflect a lack of heterogeneity in habitat quality (the composition and structure of the forest and associated suites of prey) in the essentially continuous forest cover on the Kaibab Plateau [83,122]. On the other hand, a disperser’s perceptual limitations may prevent them from discerning differences in habitat quality during prospecting excursions into large, forested territories in landscapes where prey abundance was highly variable interannually [96].

Territory occupancy can be a reliable metric of territory quality as occupancy often correlates with reproduction and other metrics of quality [66]. However, despite dispersals to territories with significantly higher occupancy rates, our data showed that dispersed hawks on average did not improve their reproductive output. This suggests that dispersal to more frequently occupied territories may be owever, Howeverbiased by the dispersals themselves. owever, However In areas where the annual territory occupancy by egg-laying pairs is as low as in our study (40%), a disperser’s departure from a territory is likely to lower that territory’s overall occupancy while raising that of the receiving territory’s. This effect is indicated by an increased change in p-values in males (Δ p = 0.393) and females (Δ p < 0.464) when the computation of the reproductive rates in the original and new territories were limited to the monitored years prior to the year of dispersal compared to when rates were determined over all monitored years, pre- and post-dispersal. This suggests that occupancy can beget occupancy irrespective of territory quality on the Kaibab Plateau.

### Habitat conservation and management

Conservation and management of goshawk habitat should focus on improving forest conditions that foster higher occupancy, reproduction, and survival [89,125,126]. Funding and capacity constraints make it necessary to identify a target subset of territories that are in the greatest need of protection or restoration. The highly variable, but overall low, annual density of breeding pairs combined with possible mismatches between territory occupancy and quality on the Kaibab Plateau, suggests an initial focus on restoring forests in the low-occupancy territories with the objective of growing the hawk population to increase occupancy and reproduction in both low- and high-fitness territories. The great majority of our study area is composed of dry conifer forest (ponderosa pine and mixed-conifer), both forest types that were historically burned by frequent low-severity surface fire (mean fire return interval, 3.0–8.7 yrs [123]). Frequent surface fire maintained a fine-scale (4 ha; [124]); mix of habitats comprised of small groups of trees with interlocking crowns separated by small grass-forb-shrub openings that, taken together, present a diversity of habitats that supported a diverse suite of goshawk prey (tree groups for tree squirrels, woodpeckers, jays, other birds; open grass-forb-shrub habitats for rabbits, hares, ground squirrels, grouse, turkeys, pigeons, other birds; [125,126]). However, decades of livestock grazing, logging, and fire suppression throughout much of the western U.S., including the Kaibab Plateau [127], allowed population explosions of young trees that invaded the grassy openings and into the tree groups resulting in closed canopies and fuel ladders. Large-tree commercial logging eliminated groups of mature and old trees and reduced the overall large-tree density. Large trees have thick fire-resistant bark and lifted live crowns and comprise important habitat for many goshawk prey species [128,129]. Restoring the fine-scale mix of habitats by removing the young trees from the openings is expected to restore the grass-forb-shrub community [130], increase the hawk’s ability to detect, pursue, and capture prey, restore the historically diverse food web on which the food-limited goshawk depends [96,108]. An added value of restoring these forests is that breaking up today’s contiguous forest canopies with grass-forb-shrub openings provides for reintroduction of low-severity surface fire and a concomitant reduction in the risk of forest-killing wildfire [131] that results in long-term loss of predator and prey habitats.

Given that goshawks in western frequent-fire forests nest and hunt within and below forests canopies with lifted crowns and into adjacent open grass-forb-shrub communities [125], we emphasize the importance and feasibility [132] of investigating the effects of restoring the natural vegetation composition and 3-dimentional heterogeneous structure of these forests on goshawk breeding density, reproduction, and survival.

## Supporting information

S1 Appendix Temporal and spatial variation in egg-laying and breeding dispersals by goshawks in Arizona, USA. (PDF)

S2 Appendix Additional summaries of territory and individual hawk metrics and parameter summary table for GAMM analysis. (DOCX)
