# Supplementary material for: Fidelity to territory and mate and the causes and consequences of breeding dispersal in American goshawk (Astur atricapillus)
Source: PLoS One. 2025 May 22;20(5):e0323805. doi: 10.1371/journal.pone.0323805 (PMC12097718; doi:10.1371/journal.pone.0323805)
Supplement: S1 Appendix — (PDF) [file pone.0323805.s001.pdf]

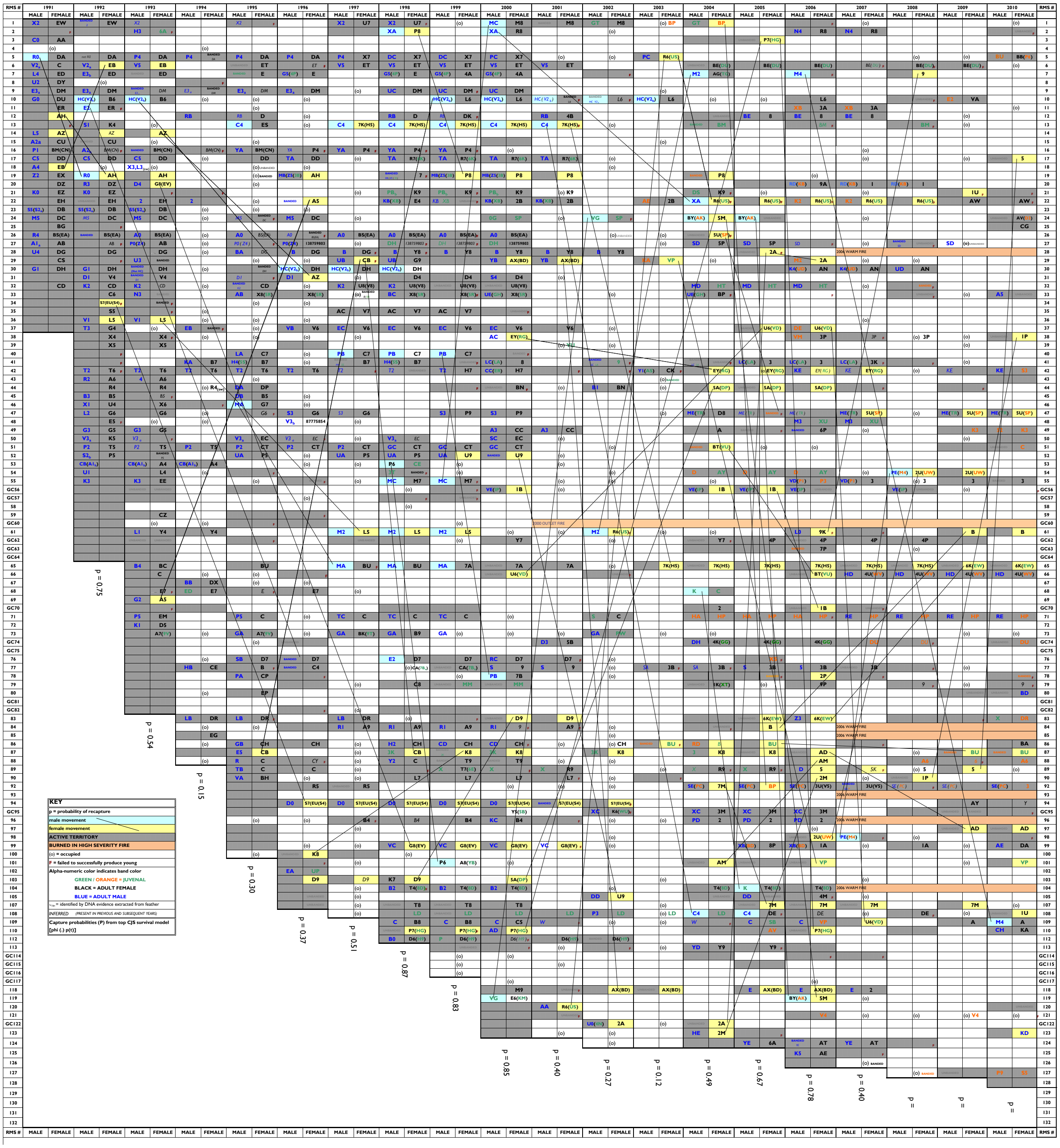

Supporting Information S1 Fig. A zoomable PDF of temporal and spatial variation in breeding (eggs laid) and local (*in situ*) breeding dispersal by American goshawks among 117 territories monitored 11 20 years on the Kaibab Plateau, Arizona, USA, 1991-2010 (to enlarge double click on figure and click on '+'). Dark cells indicate years with breeding ('occupied, eggs laid') and open cells years without egg laying (either 'occupied-only' or 'breeding status unknown'). Alpha-numeric band codes of male and female hawks in normal font indicate a year in which the identity of individual hawks was known (band code resighted). Italicized font indicates a year when hawk identities were inferred based on their prior and subsequent years of being resighted as breeders on the same territory (the bracketed rule; see Methods). Cells with a color-coded alpha-numeric followed by additional codes in parentheses indicate hawks whose color bands were replaced due to wear (difficult to read) or to exchange green and orange bands placed on nestlings for adult blue (male) and female (black) band colors. Cells with (o) were territories that contained signs of goshawk occupancy (see Methods), but no eggs were laid in that year. Lines between territories indicate breeding dispersals. Territory numbers in the far left and right columns correspond to territory numbers displayed in Fig. 1 (territories were numbered consecutively on discovery). Orange cells are territories where >64% of forest canopies within 1.9 km of the territory centroids (see Methods) were killed by high-severity fire and where breeding by goshawks was never observed again post-fire [81]. Territories 91, 106, and 111 were subsumed into other territories based on within-territory movements among alternate nests by banded breeders. Annual detection (resights) probabilities (p) of banded breeding goshawks from top CJS survival model [phi(.) p(t)] (with no sex effects) from program MARK [131] are displayed at the bottom of each year's column of territories.
