# Supplementary material for: Fidelity to territory and mate and the causes and consequences of breeding dispersal in American goshawk (Astur atricapillus)
Source: PLoS One. 2025 May 22;20(5):e0323805. doi: 10.1371/journal.pone.0323805 (PMC12097718; doi:10.1371/journal.pone.0323805)
Supplement: S2 Appendix — (DOCX) [file pone.0323805.s002.docx]

**Supporting Information S1 Table.**  Numbers of territories (*n* = 116) monitored 11 ̶ 20 years^a^, annual number of territories with active nests (eggs laid), number of territories in a prior year’s group of monitored territories, the percent of the prior year’s group of territories with active nests (a measure of the current yr’s quality for breeding), number of territories occupied-only (no eggs, but hawks or their sign observed in a nest area), number of territories with occupied status unknown (hawks or their sign not observed), and years when breeding dispersals by American goshawks were first detected on the Kaibab Plateau, Arizona, USA.

|  |  | | | | | | | | | | | | | | | | | | | | | |
| --- | --- | --- | --- | --- | --- | --- | --- | --- | --- | --- | --- | --- | --- | --- | --- | --- | --- | --- | --- | --- | --- | --- |
|  |  | 1991 | 1992 | 1993 | 1994 | 1995 | 1996 | 1997 | 1998 | 1999 | 2000 | 2001 | 2002 | 2003 | 2004 | 2005 | 2006 | 2007 | 2008 | 2009 | 2010 |  |
|  | Territories monitored | 36 | 63 | 81 | 87 | 98 | 104 | 105^a^ | 108 | 110 | 116 | 115^b^ | 115 | 115 | 115 | 115 | 115 | 109^c^ | 109 | 109 | 109 |  |
| Active nests | | 36 | 59 | 67 | 21 | 53 | 46 | 31 | 58 | 57 | 66 | 30 | 21 | 10 | 50 | 44 | 59 | 31 | 28 | 27 | 40 |  |
| Current yr quality^d^ | | - | 89 | 78 | 22 | 50 | 40 | 29 | 52 | 52 | 55 | 26 | 18 | 9 | 43 | 38 | 51 | 28 | 26 | 25 | 37 |  |
|  | Territories occupied- only | 0 | 0 | 3 | 17 | 2 | 11 | 12 | 2 | 3 | 3 | 15 | 13 | 17 | 3 | 4 | 1 | 9 | 8 | 5 | 2 |  |
|  | Territories unknown status | 0 | 4 | 11 | 49 | 43 | 47 | 62 | 48 | 50 | 47 | 70 | 81 | 88 | 62 | 67 | 55 | 69 | 73 | 77 | 67 |  |
|  | Dispersals |  |  |  |  |  |  |  |  |  |  |  |  |  |  |  |  |  |  |  |  |  |
|  | males | - | 1 | 0 | 0 | 0 | 2 | 1 | 0 | 2 | 2 | 0 | 1 | 0 | 3 | 1 | 1 | 0 | 1 | 1 | 1 |  |
|  | females | - | 2 | 0 | 0 | 0 | 3 | 2 | 2 | 2 | 1 | 0 | 3 | 2 | 3 | 6 | 8 | 1 | 2 | 4 | 3 |  |
|  |  |  |  |  |  |  |  |  |  |  |  |  |  |  |  |  |  |  |  |  |  |  |

^a^3 territories (4, 116, 117; see Fig S1) not included because they were occupied-only (never active) and 2 territories (115, 128) were not included because they were active only once with unknown breeders.

^b^1 entire territory burned by high-severity fire in 1997 Outlet Fire. Territory received standard monitoring protocol in post-fire years, but post-fire years not included in counts of monitored years because no breeding hawks were found.

^c^6 territories burned at high severity in 2006 Warm Fire, none of which had an active nest post-fire. All 20 territories partially or totally burned at high-severity in the Warm Fire (Reynolds et al. 2017) received standard monitoring protocol in post-fire years, but post-fire monitoring years not included in yearly counts of ‘territory monitored’ unless an active nest was found.

^d^Estimates of a current year’s quality for breeding, including the PYrQuality variable in the CIF, is a lagged current year quality where current year is the proportion of active nests in the group of territories discovered in the previous year (does not include territories first discovered in the current year).

**Supporting Information S2 Table.** Sample sizes of (1) total annual observations (number of sightings of territorial goshawks that were unbanded [or otherwise whose identity was unknown] + resighted banded hawks + banded hawks whose identities were inferred (see Methods) in all territories including those categorized as ‘occupied-only; (2) sample sizes and number of dispersals based on annual resight opportunities to detect dispersal (includes only banded hawks resighted in ≥2 years); (3) total annual resight opportunities of hawk pairs with complete information (i.e., mate identity known prior to dispersal opportunity) to detect mate loss or retention; and (4) total annual resight cases with complete information (i.e., original and post-dispersal mate identities known) to calculate mate quality metrics in American goshawks breeding on the Kaibab Plateau, Arizona, U.S.A., 1991-2010.

|  | Males | Females |
| --- | --- | --- |
| Total hawk observations | 905 | 948 |
| Resight opportunities to detect dispersal^a^ | 334  (*n*_dispersals_=17) | 497  (*n*_dispersals_=44) |
| Resight opportunities with known mate loss or retention^b^ | 270  (*n*_dispersals_=17) | 330  (*n*_dispersals_=34) |
| Resight opportunities with known mate quality metrics^c^ | 227  (*n*_dispersals_=13) | 212  (*n*_dispersals_=13) |

^a^ Dataset used for summary statistics.

^b^Dataset used for GAMM.

^c^Dataset used for CIF.

**Supporting Information S3 Table**. Raw percentages and (counts) of male and female American goshawks staying in an original territory or dispersing to a new territory given (1) mate retention or mate loss, (2) eggs laid or no eggs laid in ≥1 prior breeding seasons, success (fledged ≥1 young) or failure (eggs laid, no fledglings) of the prior nest attempt, and (3) combinations of nest success or failure, and mate retention or loss on the Kaibab Plateau, Arizona, U.S.A., 1991-2010. Counts within each group equals the total sample size by sex, while retained territory or dispersed outcomes are percentages for each sex in each row. Totals for (2 above) include 334 opportunities to detect retained territory or dispersed by 102 banded males and 497 opportunities by 162 banded females. Totals for (1) and (3), which have known instances of mate retention or loss, include 270 opportunities to detect territory retention or dispersal by 102 banded males and 330 opportunities by 136 banded females.

|  | | Males | | Females | |
| --- | --- | --- | --- | --- | --- |
|  | | Stayed | Dispersed | Stayed | Dispersed |
| Mate retention | | 99% (179) | 0.01% (1) | 99% (179) | 0.01% (1) |
| Mate loss | | 82% (74) | 18% (16) | 78% (117) | 22% (33) |
|  | |  |  |  |  |
| Prior failure to lay eggs | | 86% (57) | 14% (9) | 77% (85) | 23% (26) |
| Prior failure to fledge given eggs | | 83% (5) | 17% (1) | 85% (11) | 15% (2) |
| Prior successful fledgling | | 97% (255) | 3% (7) | 96% (357) | 4% (16) |
| Prior failure, no eggs | mate retention | 94% (16) | 6% (1) | 94% (16) | 6% (1) |
|  | mate loss | 76% (26) | 24% (8) | 62% (31) | 38% (19) |
| Prior failure, eggs | mate retention | 100% (4) | 0% (0) | 100% (4) | 0% (0) |
|  | mate loss | 50% (1) | 50% (1) | 75% (6) | 25% (2) |
| Prior success, eggs | mate retention | 100% (159) | 0% (0) | 100% (159) | 0% (0) |
|  | mate loss | 87% (47) | 13% (7) | 87% (80) | 13% (12) |

**Supporting Information S4 Table**. Wald tests of significance from GAMM model relating the effects of nest outcome: (1) no eggs produced in ≥1 prior breeding seasons; (2) eggs produced in the prior nest attempt but nest failed; (3) nest success [fledglings produced] in the prior nest attempt), (4) whether the prior mate was retained at the next nest attempt, (5) interaction of sex with a non-linear effect of territory familiarity (yrs a hawk was known and inferred to be in a territory; see Methods) to be in its territory, and (6) a random effect for territory on breeding dispersal by American goshawks on the Kaibab Plateau, Arizona, USA, 1991-2010. Observations include 600 resights (including inferred territory and mate retentions) by 238 banded goshawks in 87 territories.

|  |  | df or edf | $Test statistic$ | p-value |
| --- | --- | --- | --- | --- |
| Linear terms | mate retention | 1 | 19.15 | 0.0000121 |
|  | nest outcome | 2 | 21.82 | 0.0000183 |
| Non-linear terms | territory familiarity:male | 1.31 | 2.15 | 0.35 |
|  | territory familiarity:female | 1.45 | 12.71 | 0.004 |
| Random effect | territory | 0.81 | 4.39 | 0.02 |

**Supporting Information S2 Fig**

**
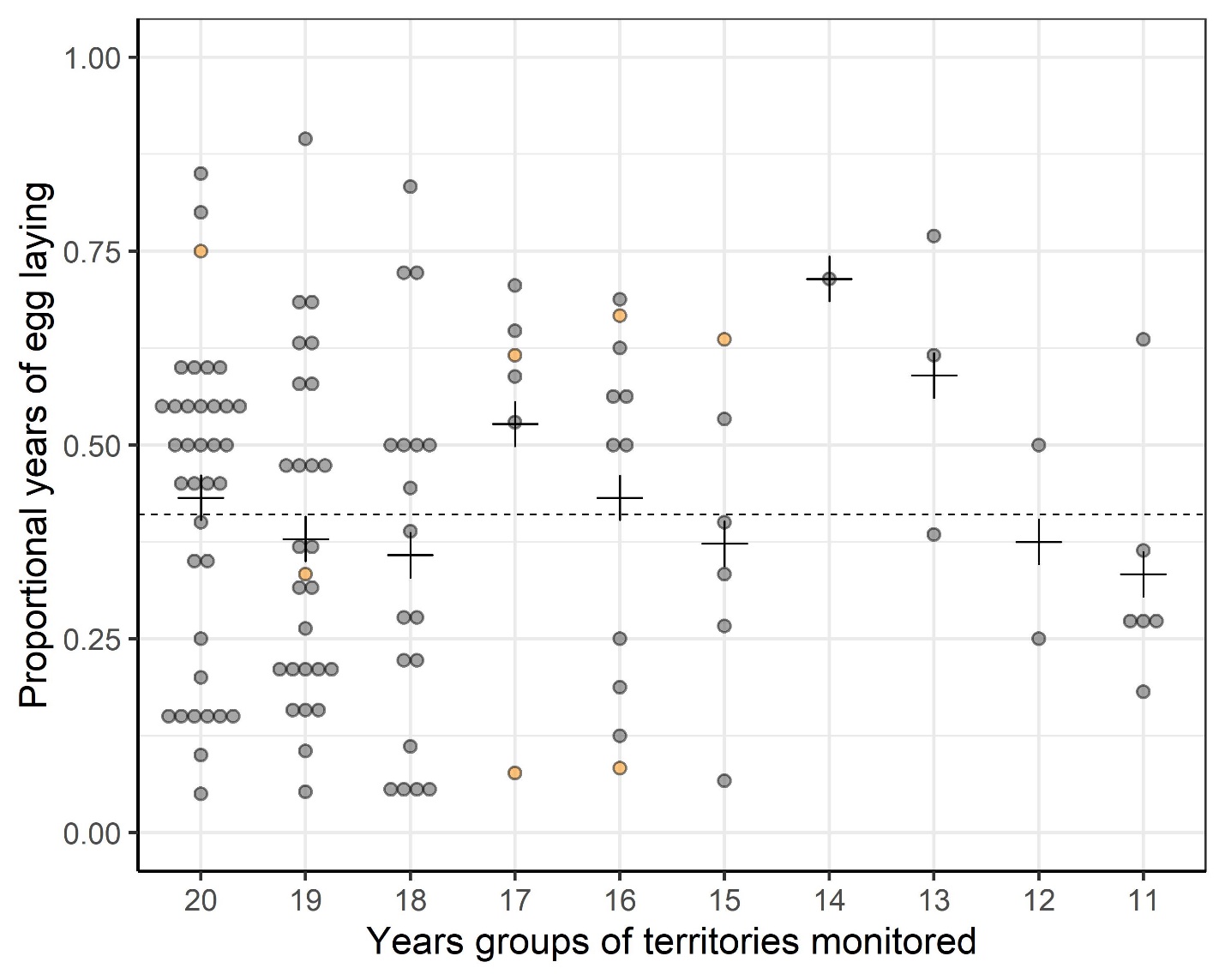
**

**Supporting Information S2 Fig.** Does the proportion of territories occupied by breeding (eggs-laid) American goshawks vary by the year of territory discovery? The means and variation in the proportions of monitored years that 116 territories were occupied by egg laying American goshawks in 10-territory groups based on their year of discovery and monitored 11-20 years on the Kaibab Plateau, Arizona, USA, 1991-2010. Horizontal line is the overall proportional mean (0.40, range = 0.09-0.89) of years all territories were occupied by egg-laying breeders. The + symbols are the mean proportion of years of occupancy by each territory group. Years of occupancy of 7 territories (yellow dots) were adjusted to account for loss of forest habitats to high-severity fire (see Fig. 1). Five territories (4, 115, 116, 117, 128) not included as banded goshawks on these territories never laid eggs.

**Supporting Information S3 Fig**


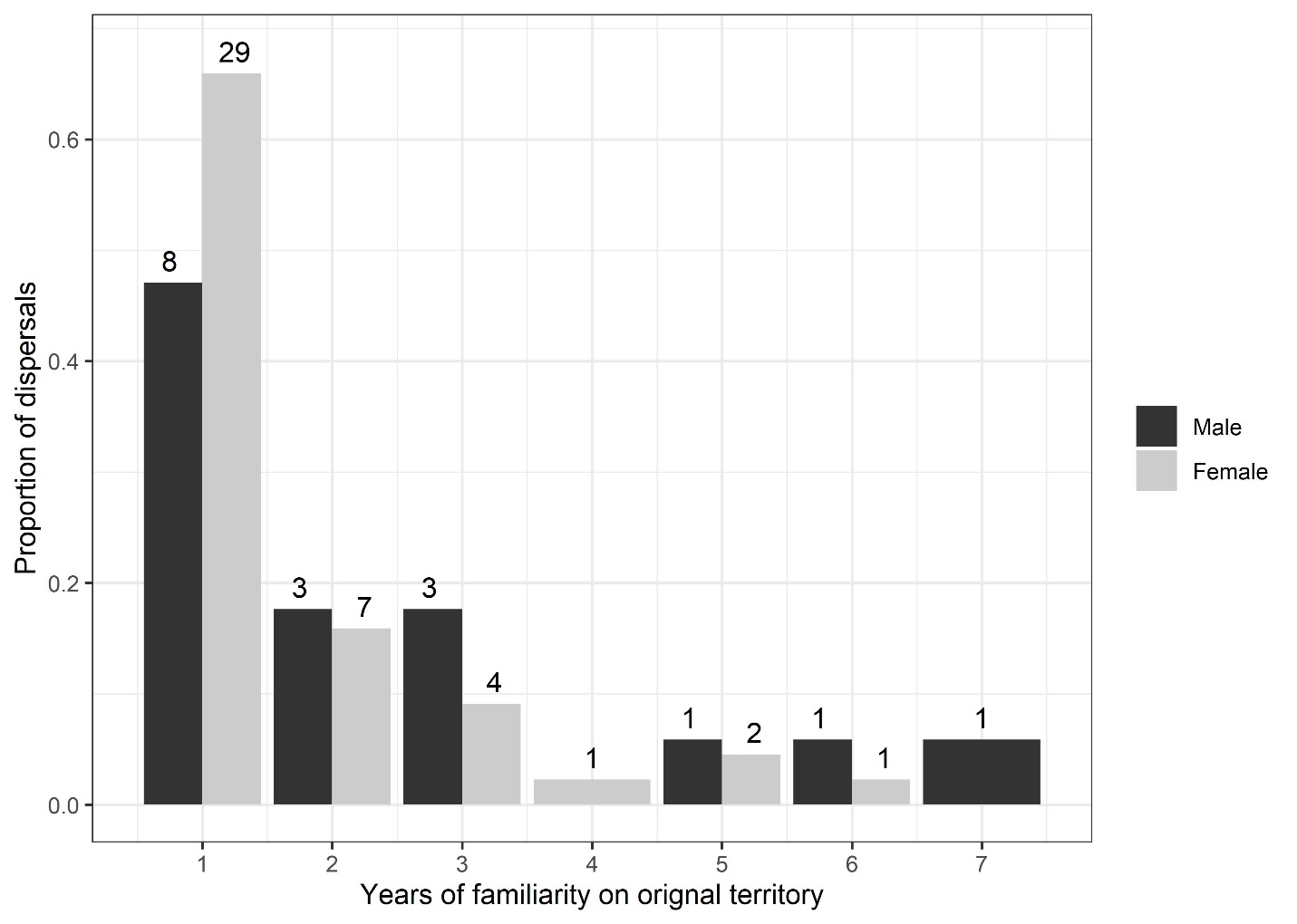


**Supporting Information S3 Fig**. The number of years (1^st^ to last breeding attempt) of familiarity with their original territory prior to dispersal by 17 male and 44 female American goshawks on the Kaibab Plateau, Arizona, USA, 1991‒2010. Counts of hawks in each category are displayed above bars and category proportions were derived from the 17 dispersed males and 44 dispersed females.

**Supporting Information S4 Fig**
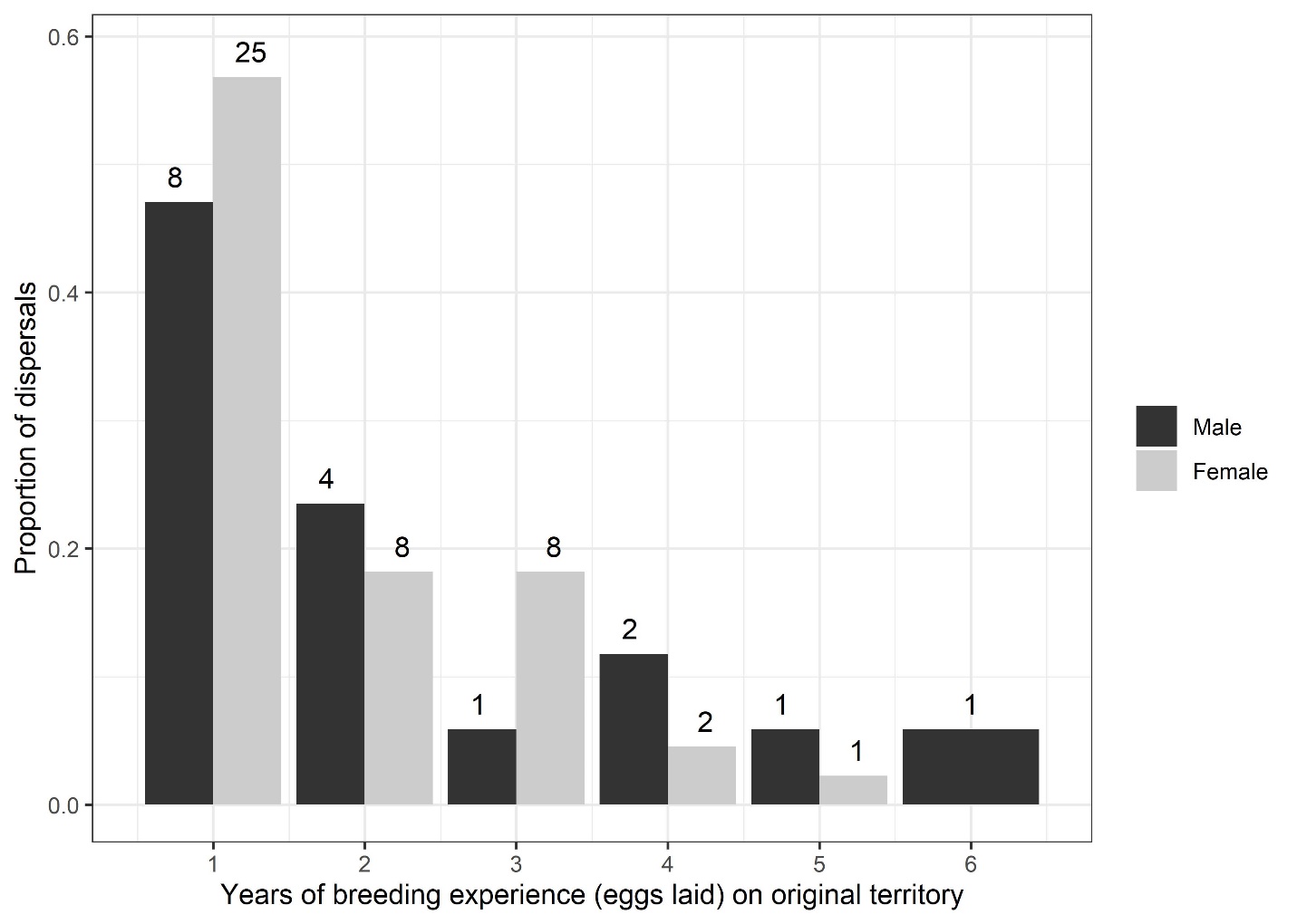


**Supporting Information S4 Fig.** The number of years of breeding experience (yrs of egg laying) in their original territory prior to dispersal by 17 male and 44 female American goshawks on the Kaibab Plateau, Arizona, USA, 1991‒2010. Counts of hawks in each category are displayed above bars and category proportions were derived from numbers of dispersed males and females separately.

**Supporting Information S5 Fig**


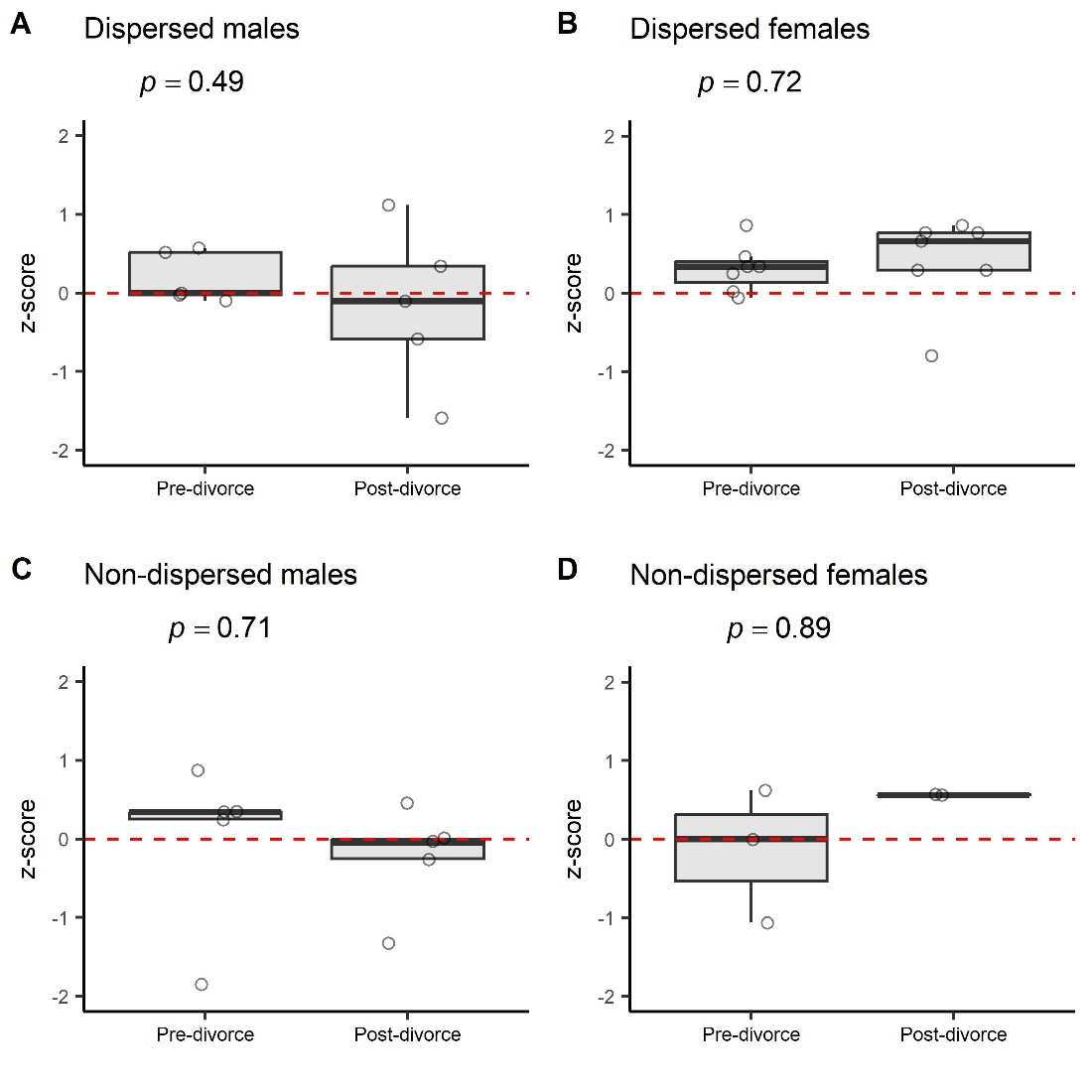


**Supporting Information S5 Fig**. Did the production of fledglings by divorced male and female American goshawks improve after divorce? Distribution of the differences (before ‒ after) in mean annual *z*-scores of fledgling production before and after divorce (*n* = 10) for (A) male and (B) female American goshawks that dispersed, and (C) males and (D) females that stayed in their territories post-divorce on the Kaibab Plateau, Arizona, USA, 1991-2010. Red horizontal line indicates equal productivity before and after divorce, positive values show higher productivity after divorce, and negatives show lower productivity after divorce.

**­­­­­­­­­­­­­­­­­­­­­**
